# Nanozymes in Point-of-Care Diagnosis: An Emerging Futuristic Approach for Biosensing

**DOI:** 10.1007/s40820-021-00717-0

**Published:** 2021-09-13

**Authors:** Bhaskar Das, Javier Lou Franco, Natasha Logan, Paramasivan Balasubramanian, Moon Il Kim, Cuong Cao

**Affiliations:** 1 School of Biological Sciences, Queen’s University Belfast, Belfast, UK; 2 Department of Biotechnology and Medical Engineering, National Institute of Technology Rourkela, Rourkela, India; 3 Department of BioNano Technology, Gachon University, Seongnam, Korea

**Keywords:** Nanozymes, Biosensing, Point-of-care diagnosis, ASSURED diagnostics, Catalytic nanomaterials

## Abstract

Nanomaterial-based artificial enzymes (or nanozymes) have attracted great attention in the past few years owing to their capability not only to mimic functionality but also to overcome the inherent drawbacks of the natural enzymes. Numerous advantages of nanozymes such as diverse enzyme-mimicking activities, low cost, high stability, robustness, unique surface chemistry, and ease of surface tunability and biocompatibility have allowed their integration in a wide range of biosensing applications. Several metal, metal oxide, metal–organic framework-based nanozymes have been exploited for the development of biosensing systems, which present the potential for point-of-care analysis. To highlight recent progress in the field, in this review, more than 260 research articles are discussed systematically with suitable recent examples, elucidating the role of nanozymes to reinforce, miniaturize, and improve the performance of point-of-care diagnostics addressing the ASSURED (affordable, sensitive, specific, user-friendly, rapid and robust, equipment-free and deliverable to the end user) criteria formulated by World Health Organization. The review reveals that many biosensing strategies such as electrochemical, colorimetric, fluorescent, and immunological sensors required to achieve the ASSURED standards can be implemented by using enzyme-mimicking activities of nanomaterials as signal producing components. However, basic system functionality is still lacking. Since the enzyme-mimicking properties of the nanomaterials are dictated by their size, shape, composition, surface charge, surface chemistry as well as external parameters such as pH or temperature, these factors play a crucial role in the design and function of nanozyme-based point-of-care diagnostics. Therefore, it requires a deliberate exertion to integrate various parameters for truly ASSURED solutions to be realized. This review also discusses possible limitations and research gaps to provide readers a brief scenario of the emerging role of nanozymes in state-of-the-art POC diagnosis system development for futuristic biosensing applications.

## Highlights

- Enzyme-mimicking activities of different nanomaterials (nanozymes) and the recent progress in the construction of nanozyme-based biosensors with various examples are discussed in this review.
- Physicochemical properties of nanomaterials (size, composition, pH, temperature, surface chemistry) play crucial role in the nanozyme activities.
- The emerging nanozyme-based biosensors promise great potential for point-of-care diagnostic applications following the ASSURED criteria defined by WHO.

## Graphical abstract

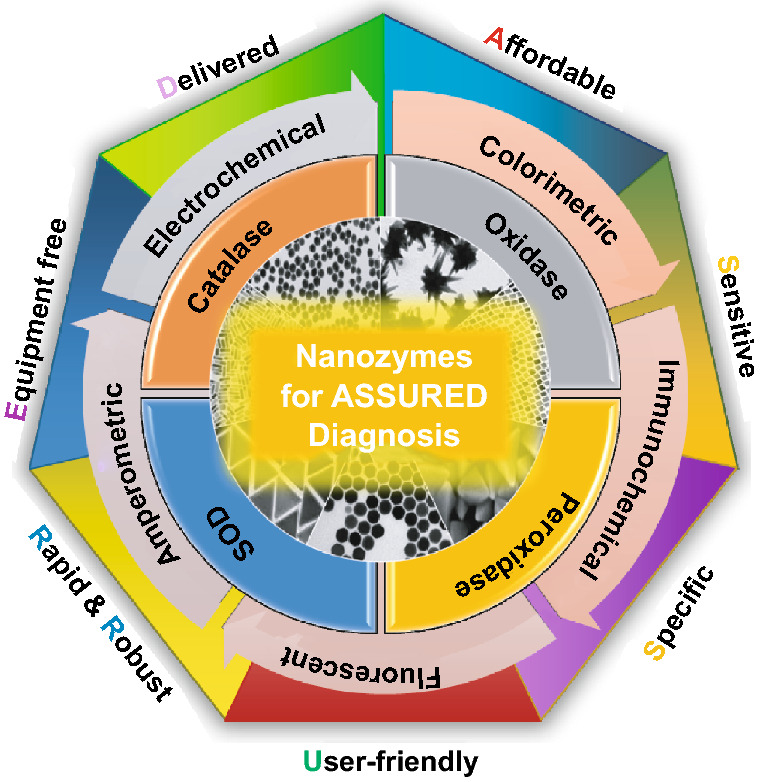

## Introduction

**Fig. 1:**
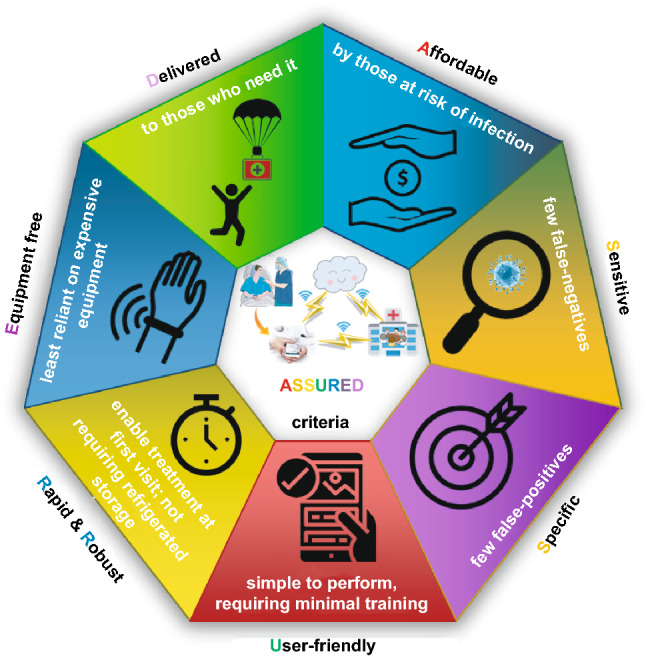
World Health Organization’s ASSURED criteria for point of care device

In the last decade, early diagnosis of disease has become an emerging area of exploration for the worldwide medical community. Recent advancements made in medicine have played an immense role in maintaining the standards of healthcare and wellbeing. However, despite the efforts we have witnessed that late and inappropriate diagnosis is still the main causes of death. During the period 1990–2004, it was estimated that 40% of the annual deaths worldwide were caused by infectious diseases [1]. The early detection of diseases leads to ease in the therapeutic decision-making, along with decreasing the severity and lethality of the diseases. Therefore, to prevent these disease outbreaks and to facilitate the early diagnosis, there has been a drive to develop various advanced biosensing technologies for sensitive and specific detection of biomarkers, the naturally occurring biomolecules that represent disease status. Accordingly, detections of bio-threat agents, chemical contaminants, toxins, biomolecules, and pathogens have attracted much attention, and numerous conventional technologies such as real-time polymerase chain reaction (RT-PCR), Enzyme-linked immunosorbent assay (ELISA), DNA microarrays, immunosensors, spectroscopic and spectrometric techniques have been employed [2]. Although these potential conventional technologies offer specific and sensitive detection of disease biomarkers or pathogens, they still lack behind in providing a sweeping solution for the disease outbreaks due to at least one or all of these reasons: the privation of accessibility and affordability for the high-cost equipment facilities in the remote locations of the developing countries, time-consuming processes, complicated sample pre-treatment, and the need for technical expertise [3]. Hence, the development of low-cost, innovative Point-of-care (POC) testing for detection and diagnosis has become the need of the hour in the field of biosensor and bioassay development. POC systems have been proven to be particularly useful in resource-poor areas to deal with disease outbreaks. However, the diagnostic systems have to follow guidelines formalized by World Health Organization (WHO), which involves ASSURED criteria (described in Fig. 1): affordable, specific, sensitive, user-friendly, rapid and robust, equipment-free, and delivered to the end users.

**Fig. 2:**
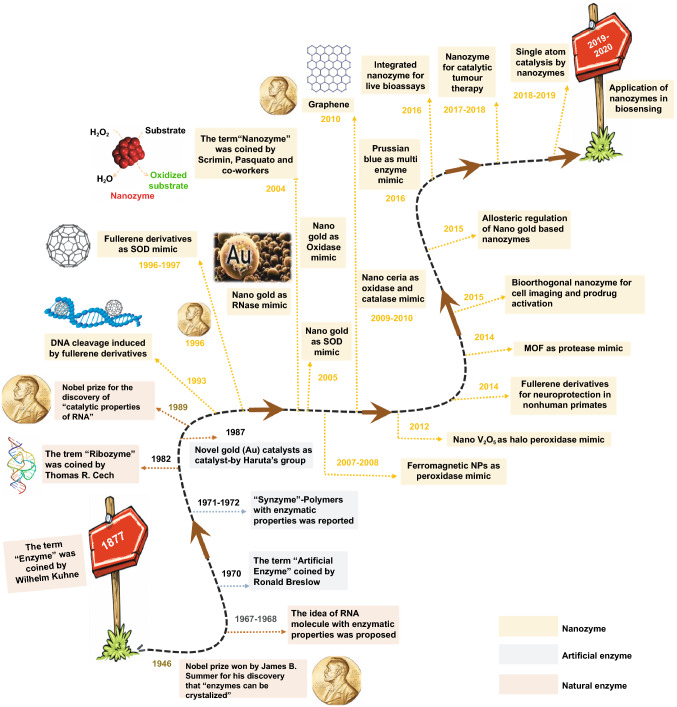
A brief timeline of the development of nanozymes over the years (natural enzymes and artificial enzymes are listed for comparison)

Natural enzymes are biological catalysts that convert substrates into products in biochemical processes. The term “enzyme” was first coined by Wilhelm Kühne in 1877. After that in 1926, James B. Sumner discovered that the first enzyme, urease, was a protein [4], which won him the Nobel Prize in 1946 (Fig. 2). Natural enzymes have been considered proteins and utilized extensively as one of the key components in POC biosensor development due to their highly efficient biocatalytic activity which can catalyse a range of specific chemical or biochemical reactions resulting in generation of colorimetric or electrochemical signals in the presence of analytes with optimum performance and substrate specificity. For example, glucose oxidase [5], alcohol oxidase [6] lactate oxidase [7], lactate dehydrogenase [8], cholesterol oxidase [9], urate oxidase [10], horseradish peroxidase (HRP) [11], cytochrome c reductase [12] are those natural enzymes which have been utilized in many biosensing applications. However, the natural enzymes possess some characteristics that limit their biosensing applicability in a broader range: restricted physiological working conditions, reduced stability in harsh environments, high production costs, and complexity of production process. Therefore, over the past few years, there has been a surge of efforts to explore enzyme mimics or “artificial enzymes”, a term coined by Ronal Breslow in 1970 [13], or alternatives to natural enzymes such as protein enzymes, catalytic ribozymes (i.e. DNAzymes, RNAzymes). The catalytic properties of RNA or the term “ribozyme” was first coined by R. Cech which won him the Nobel Prize in 1989 (Fig. 2) [14]. So far, researches in the field have demonstrated that synthetic enzymes can play an important role in the field of biomimetic chemistry, showcasing potentials in bionics, biosensing and biomedical applications [15–17]. Early work pioneered by Haruta’s group in 1987 led to the discovery that novel gold (Au) catalysts could be employed to catalyse the oxidation of carbon monoxide at temperatures as low as -70 °C [18]. It was this research conducted by Haruta’s group during the 1980s which led to the discovery (2004) that gold nanoparticles (Au NPs) could act as biological catalysts (oxidase mimic, RNase mimic) due to their high surface-area-to-volume ratio. This important discovery paved the way for extensive interest and research in the area to understand the catalytic properties of Au, which was historically considered as chemically inert.

Furthermore, recent advancements in the area of nanotechnology have led to considerable growth in the development of functional nanomaterials that mimic activities of the natural enzymes (nanozymes) (a brief timeline is shown in Fig. 2). Scrimin, Pasquato and co-workers coined the term “nanozymes” for the first time in 2004 while describing their thiol protected gold nanoclusters with excellent ribonuclease mimicking activity [19]. Later, nanozymes were defined as 'nanomaterials with intrinsic enzyme-like activity’, due to their ability to mimic the actions of natural enzymes, by replicating kinetic behaviours and catalysing the conversion of substrates to oxidized coloured products (a brief time line of the evolution of different enzyme-mimicking nanomaterials and their field of applications since the discovery of nanozymes is shown in Fig. 2) [14]. The nanozymes exhibit significant advantages such as unflinching biocatalytic activity, ultrahigh environmental stability as inorganic materials making them unaffected by biological degradation activities, excellent robustness, facile synthesis and functionalization and low-cost production [20–22]. Typical enzyme-mimicking functional nanomaterials include metals [23], metal oxides [24], metal chalcogenides [25], nanocarbon materials [26], layered double hydroxides (LDHs) [27], and metal–organic frameworks (MOFs) [28]. Additionally, most of these nanozymes are capable of mimicking natural enzymes in four main different enzymatic reactions, namely catalase [29], peroxidase [30], oxidase [31], and superoxide dismutase (SOD) [32] for the oxidation of chromogenic substrates, e.g. 3,3'-diaminobenzidine (DAB) [33], 2,2'-Azino-bis(3-ethylbenzothiazoline-6-sulphonic acid) (ABTS) [34], and 3,3’,5,5’-tetramethylbenzidine (TMB) [35]. Once oxidized, each substrate can result in coloured aqueous solutions, which can be analysed by the naked eye, and a unique absorption spectrum detected using a spectrophotometer.

The nanozymes, owing to these enzymatic characteristics, have been exploited in a wide range of applications within various research fields such as biosensing, immunoassays, pollutant removal, disease diagnostic, and therapeutic [22]. Therefore, biocatalytic properties of the enzyme-mimicking nanomaterials have been an imperative part of the fabrication of point-of-care devices. The explicit robustness, low cost, and high stability for field application make the nanozymes more suitable for utilization as a transducing element in POC devices following the WHO’s guidelines of ASSURED criteria (Fig. 1) which makes them more affordable, specific, robust, and easy to use in the resource-limited area of developing countries. This review covers a systematic literature assessment of more than 260 research articles based on the recent developments and the progress of nanozymes in biosensing applications during the period of past 15 years up to March 2021. The review also indicates how the flexible characteristics of nanozymes like facile synthesis, robust nature, ease of surface modulation, and wide range of catalytic properties depending on physicochemical parameters (*e.g.* pH, material composition, size, and shape) assess their propriety for biosensing applications. Although there are few review studies have been published earlier illustrating the enzyme-mimicking characteristics of different nanomaterials with their potential biosensing applications [22, 36–39]. However, to the best of our knowledge there is no such systematic review study available in the scientific database explaining the suitability of different nanozymes in the field of biosensing which has the potential applicability at POC settings following the ASSURED criteria of WHO. Moreover, various types of developed biosensing strategies consisting of nanozymes as an integral part of the sensing mechanism following each of the criteria are described separately with relevant examples. A brief introduction of different enzyme-mimicking characteristics of a broad range of nanomaterials is also discussed in this study. The major focus of this review is emphasized on highlighting the advantageous characteristics of nanozymes enabling their aptness for POC-based biosensor development with possible limitations and research gaps to provide readers a brief scenario of the emerging role of nanozymes in state-of-the-art POC diagnosis system development for futuristic biosensing applications.

## Different Nanomaterials with Enzyme-like Characteristics for Biosensing Applications

**Fig. 3:**
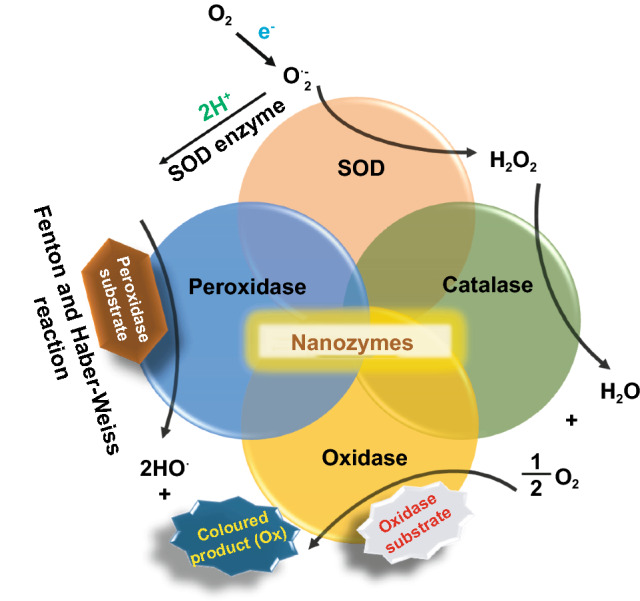
Schematic representation of different enzyme-mimicking activities by nanozymes in the presence of superoxide anions produced by single electron donor

Conventional enzyme-based biosensing mechanism mostly relies on the catalytic efficiency of various natural enzymes as mentioned in the earlier section. For example, ELISA-based biosensors utilize HRP for the catalytic oxidation of TMB or ABTS, thereby generating a colorimetric signal for qualitative and quantitative detection of target analytes [40]. However, due to the limited biosensing applicability of these natural enzymes, researches have been concerted for the development of suitable artificial enzymes in last two decades. During the past five years, aiding by the rapid improvement of nanotechnology, biotechnology, catalysis science, and computational designs, enormous advances have been accomplished in reproducing new enzymatic activities with highly efficient nanomaterials, managing the nanozyme activities, explaining the catalytic mechanism, and widening possible applications. Based on the recent findings on nanomaterials endowing excellent catalytic efficiency and enzyme-mimicking activity, several nanozymes have been utilized by many researchers for the development of POC-based biosensing systems for real-time applications. For better understanding of the recent progress on nanozyme-based biosensor developments, we briefly summarize a list of different nanomaterials in Table 1 along with their type of enzyme-mimicking activities, sensing mechanism, target analyte, and their limit of detection for the potential POC-based biosensing applications. In general, applications of nanozymes based on their enzyme-mimicking activity can be classified into four major groups (Fig. 3): peroxidase, oxidase, catalase, SOD mimics [41]. Numerous nanomaterials with these enzyme-mimicking activities and their mechanism for biosensing applications are discussed in this section with recent suitable examples.

**Table 1:** Different enzyme-mimicking nanomaterials used for biosensing applications

Nanomaterial	Enzyme activity	Target	Type of sensor	LOD	References
Au@PtNP/GO	Peroxidase	H_2_O_2_	Colorimetric	1.62 µM	[120]
Fe_3_O_4_	Peroxidase	Ebola virus	Colorimetric	1 ng mL^−1^	[121]
Au@Pt nanorods	Peroxidase	Rubella virus	Colorimetric	10 ng mL^−1^	[122]
Au NPs	Peroxidase	Human norovirus	Colorimetric	200 viruses mL^−1^	[123]
Cobalt oxyhydroxide nanoflakes (CoOOH NFs)	Peroxidase	Acetylcholinesterase	Colorimetric	33 mU mL^−1^	[124]
Fe_3_O_4_-Pt/ core–shell	Peroxidase	Human chorionic gonadotropin (hCG)	Colorimetric (paper-based strip)	0.025 ng mL^−1^	[125]
PtNPs	Peroxidase	Uric acid	Colorimetric	4.2 µM	[126]
(BSA)−Cu_3_(PO_4_)_2_·3H_2_O	Peroxidase	Acetylcholinesterase	Colorimetric	0.0005 mM	[127]
Pt NPs	Peroxidase	Acute-phase HIV in clinical human plasma samples	Colorimetric (paper-based strip)	ca. 0.8 pg mL^−1^	[128]
Cu HDS nanoflowers	Peroxidase	Glucose	Colorimetric	0.5 μM	[129]
Pd@Pt NPs	Peroxidase	*Salmonella Enteritidis* and *Escherichia coli* O157:H7	Colorimetric	∼20 CFU mL^−1^ for *S*. e*nteritidis and ∼34* CFU mL^−1^ *for E. coli O157:H7*	[130]
Fe-MOF-Au NPs	Peroxidase	*Salmonella Enteritidis*	Colorimetric immunosensor	∼34 CFU mL^−1^	[131]
H-Pt NPs	Peroxidase	Immunoglobulin E (IgE)	Colorimetric immunosensor	0.17 kU L^−1^	[132]
Pd–Pt NPs	Peroxidase	*Escherichia coli* O157:H7	Colorimetric paper-based sensor	9.0 × 10^2^ CFU mL^−1^	[133]
Au–NPFe_2_O_3_ nanocomposites (NC)	Peroxidase	p53-specific autoantibodies	Electrocatalytic and colorimetric	0.08 U mL^−1^	[134]
Janus γ-Fe_2_O_3_/SiO_2_ nanoparticles	Peroxidase	Glucose	Colorimetric	10.6 nM	[135]
Cu/Au/Pt trimetallic nanoparticles	Peroxidase	Glucose	Colorimetric	H_2_O_2_: 17 nM, Glucose: 33 mM	[136]
Fe_3_O_4_	Peroxidase	Glucose	Colorimetric	3 μM	[137]
Nickel–palladium hollow nanoparticles (NiPd hNPs)	Peroxidase	Glucose	Colorimetric	4.2 μM	[138]
Fe_3_O_4_	Peroxidase	*Listeria monocytogenes*	Colorimetric	5.4 × 10^3^ CFU mL^−1^	[139]
Cu (II)-based metal–organic xerogels	Peroxidase	Dopamine	Colorimetric	85.76 nM	[140]
Fe_3_O_4_	Peroxidase	Prostate-specific antigen	Photoelectrochemical (PEC) immunoassay,	18 fg mL^−1^	[141]
Fe_3_O_4_	Peroxidase	Micro RNA	Electrochemical	33 aM	[142]
CoOxH-GO nanohybrid	Peroxidase	Glucose and cyanide ions	Colorimetric biosensor	Glucose: 32 nM CN ions: 10 μM	[143]
MnO_2_ nanosheets	Peroxidase	Glutathione (GSH)	Colorimetric	300 nM	[144]
Pd@AuNRs	Peroxidase	Malathion	Colorimetric	60 ng mL^−1^	[145]
Co_3_O_4_/CuO hollow nanocage	Peroxidase	Dopamine	Colorimetric	0.05 μM	[146]
Mesoporous carbon-dispersed Pd NPs	Peroxidase	H_2_O_2_	Colorimetric smart phone-based (POC)	0.05 μM	[147]
H‐Pt NPs	Peroxidase	hcG	Colorimetric LFIA, POC	0.2 ng mL^−1^	[125]
Fe_3_O_4_	Peroxidase	Hepatitis B virus surface antigen (preS1)	Colorimetric, Immunoassay	–	[50]
Au@Pt NPs	Peroxidase	PSA antigen	Colorimetric, Immunoassay paper-based strip (LFD)	3.1 pg mL^−1^	[148]
Cu_3_(PO_4_)_2_	Peroxidase	Glucose	Colorimetric	25 μM	[149]
γ-MnOOH nanowires	Peroxidase	AChE, omethoate, dichlorvos	Colorimetric paper-based (POC)	AChE: 0.1 mU mL^−1^ Omethoate: 10 ng mL^−1^ Dichlorvos: 3 ng mL^−1^	[150]
CuO/WO_3_-GO	Peroxidase	Cancer cells	Electrochemical biosensor, POC	18 cells mL^−1^	[151]
Co_3_O_4_-CeO_2_ nanosheets	Peroxidase	Glucose	Colorimetric, POC	0.21 μM	[152]
Co_2_(OH)_2_CO_3_-CeO_2_	Peroxidase	Carcinoembryonic antigen (CEA)	Colorimetric, POC smart phone	0.51 pg mL^−1^	[153]
MnCo_2_O_4_	Peroxidase	Ochratoxin A (OTA)	Colorimetric	0.08 ng mL^−1^	[154]
CeO_2_	Peroxidase	Glucose in serum	Fluorescence	130 nM	[155]
CePO_4_ − CeO_2_ composite nanorods	Peroxidase	H_2_O_2_, glucose	Colorimetric	H_2_O_2_: 2.9 μM Glucose: 4.1 μM	[156]
Fe@PCN-224 NPs	Peroxidase	Glucose	Colorimetric	22 μM	[157]
MoS_2_ 2D‐nps	Peroxidase	Lipase detection	Colorimetric	5 nM	[158]
CeO_2_ (Nanoceria)	Oxidase	Dopamine, catechol	Colorimetric	Dopamine: 1.5 μM Catechol: 0.2 μM	[159]
CeO_2_	Oxidase	Prototypic cancer biomarker (EpCAM)	Fluorescence	–	[77]
Ag nanoclusters	Oxidase	Hg^2+^ and DNA	Colorimetric	Hg^2+^: 25 nM DNA: 10 nM	[160]
MnO_2_ nanorods	Oxidase	Hg^2+^	Colorimetric	0.08 μM	[161]
Cit-AgNPs	Oxidase	Hg^2+^	Colorimetric	2.8 × 10^–8^ M	[162]
Au@PtNP	Catalase (decomposition of H_2_O_2_)	Cocaine	Pressure-based sensor	0.06 μM	[163]
PtPd NPs	Catalase	Butyrylcholinesterase (BChE)	Colorimetric	0.05 nM	[164]
Pt NPs and carbon nanotube	Catalase	Carcinoembryonic antigen (CEA)	Pressure-based sensor with immunoassay and digital multimeter	167 pg mL^−1^	[165]
CeO_2_	Catalase	H_2_O_2_	Fluorescence	0.15 μM	[166]
Au NPs	SOD, catalase	Potential biological effects of pH	Electron spin resonance (ESR)	–	[101]
Au@Pt NPs	Catalase	Regulation of hypoxic TME and enhance cell mediated anti-tumour immunity	Fluorescence, O_2_ generation, immunoassay	–	[89]
Au NPs	SOD, catalase	Potential biological effects of pH	Electron spin resonance (ESR)	–	[101]
Au@Pt NPs	Catalase	Regulation of hypoxic TME and enhance cell mediated anti-tumour immunity	Fluorescence, O_2_ generation, immunoassay	–	[89]
AgNPs	Catalytic reduction	H_2_O_2_, Hg^2+^	Colorimetric	H_2_O_2:_10 nM Hg^2+^: 2 nM	[167]
Pt/Au NPs	Peroxidase, catalase	Hg^2+^ and MeHg	Fluorescence	Hg^2+^: 2.5 nM MeHg: 4 nM	[168]
Iron phosphate microflowers	Peroxidase, SOD	H_2_O_2_	Colorimetric	–	[169]
DNA-Ag/Pt nanoclusters	Inverse peroxidase	Hg^2+^	Colorimetric	5 nM	[170]
BSA-AuNCs	Peroxidase	Cysteine, Hg^2+^	Colorimetric	Cysteine: 80 nM Hg^2+^: 30 nM	[171]
BSA-stabilized Au clusters	Peroxidase	Xanthine and H_2_O_2_	Colorimetric	Xanthine: 5 × 10^–7^ M H_2_O_2_: 2 × 10^–8^ M	[65]
AuNP/Ag-bipyridine hybrids	Peroxidase	H_2_O_2_, D-glucose	Electrochemical	H_2_O_2_: 10 µM D-glucose: 100 μM	[34]
rGO/PEI/Pd nanohybrids	Peroxidase	Hg^2+^	Colorimetric	0.39 nM	[172]
Au NPs	Peroxidase	Melamine	Colorimetric	0.2 nM (by UV–Vis) 0.5 µM (by naked-eye)	[35]
Cyst-Au NPs	Peroxidase	Sulphate	Colorimetric	0.16 µM (by UV–Vis) 4 µM (by naked eye)	[173]
Aptamer-functionalized Au NPs	Peroxidase	Thrombin	Colorimetric	0.02 pM	[174]
Sphere-like CoS	Peroxidase	H_2_O_2_, Hg^2+^	Colorimetric	H_2_O_2_: 0.02 mM Hg^2+^: 0.1 µM	[175]
Kiwi juice-capped Au NPs	Peroxidase	Cysteine	Colorimetric	6.2 × 10^–9^ M	[176]
Au NPs	Peroxidase	Hg^2+^	Colorimetric	1.45 nM	[177]
Au NPs	Peroxidase	Metal ions (Pb^2+^ and Hg^2+^)	Fluorescence	Pb^2+^: 1.6 nM Hg^2+^: 1.2 nM	[178]
Au NPs	Peroxidase	Antibiotics	Colorimetric	86 nM	[179]
Chitosan-PtNPs	Peroxidase	Biomarkers	Colorimetric	0.016 U L^−1^	[31]
Pt-NPs	Peroxidase	DNA	Colorimetric	0.4 nM	[180]
Positively charged Au NPs	Peroxidase	Glucose	Colorimetric	4 × 10^–6^ M	[64]
Au NPs	Peroxidase	Potassium ions (K^+^)	Colorimetric	0.06 nM	[181]
AuNP-carbon NT hybrid	Peroxidase	Influenza virus	Colorimetric	3.4 PFU mL^−1^	[182]

### Peroxidase-Mimicking Nanozymes

Peroxidases are natural enzymes consisting of a large family of enzymes, which are capable of oxidizing peroxidase substrates in the presence of hydrogen peroxide (in some cases with organic hydrogen peroxides). Owing to their excellent catalytic properties, peroxidase enzymes are of considerable importance in biological systems. They are proficient of detoxifying free radicals (*e.g*. glutathione peroxidase) and enabling defence against the invading pathogens (*e.g*. myeloperoxidase) [42]. Further, peroxidases (*e.g.* HRP) have been widely exploited in the field of clinical and bioanalytical chemistry for enzymatically driven catalysis of colorimetric substrates leading to generate signals for detection of target analytes. In recent years, many review studies have shown that specific nanomaterials are efficient of exhibiting peroxidase-like activities. Most widely accepted mechanism behind the peroxidase-like activity of nanomaterials (metal, MOFs) adopted under acidic conditions, where the activated H_2_O_2_ act as an electron donor and undergoes alkaline decomposition on the surface of the nanomaterials which further assisted the production of OH and oxygen superoxide anion (O_2_^·¯^). The ·OH and O_2_^·¯^ radicals would induce the subsequent oxidation of the peroxidase substrates resulting in formation of coloured products [22, 43, 44]. Interestingly, in special cases, the peroxidase-like activity of precious metals such as gold, platinum, palladium depends on “one-step electron transfer mechanism”. For example, the catalytic mechanism of negatively charged Au nanomaterials has been reported to be similar to that of HRP, which can be explained by the decomposition of H_2_O_2_, more specifically hydrogenation by H_2_ and oxidation by O_2._ The possession of an extra electron from Au can be readily transferred to adsorbed-O_2_ on the surface of the Au NPs which in turn weakens the O–O bond. This leads to H_2_O_2_ being split into double hydroxyl radicals which can be stabilized by Au via a partial electron exchange interaction (one-step electron transfer). The newly formed hydroxyl radicals oxidize TMB, thus contributing to the nanozymes’ peroxidase-like activity [45]. Furthermore, negatively charged Au has been reported to have a higher catalytic activity and greater affinity to TMB substrate than positively charged Au, e.g. amino-modified Au [46]. This is due to electrostatic interactions between the positive charges of TMB substrate and the negative charges of the citrate-capped AuNPs, which is thought to contribute further to their enzyme-mimicking behaviour. A schematic illustration of peroxidase-mimicking catalytic activity of nanozymes is shown in Fig. 3.

Besides, the above-mentioned pathways behind the peroxidase activity, catalytic mechanisms of various nanomaterial-based peroxidase mimics could commonly be determined as Fenton or Fenton-like reaction or the electron transfer process [47] (Fig. 3). It is well reported that H_2_O_2_ can be converted into highly reactive hydroxyl radicals in the presence of metal ions (specially Fe^2+^) via “Fenton reactions” [48]. This phenomenon was first discovered by H.J.H. Fenton in 1894 who proposed that several metals have special oxygen transfer properties which improve the use of H_2_O_2_. Besides the Fenton reaction, in the presence of strong catalytic metal ions (mostly iron ions) H_2_O_2_ can be converted into reactive ·OH (hydroxyl radicals) and superoxide anion (·O_2_^−^) via “Haber–Weiss reactions” [49], which was first proposed by Fritz Haber and his student Joseph Joshua Weiss in 1932. A schematic illustration of peroxidase-mimicking catalytic activity of nanozymes via Fenton reaction is shown in Fig. 3. In 2007, Yan and co-workers first demonstrated the peroxidase-like activity of iron oxide (Fe_3_O_4_) magnetic nanoparticles (MNPs) via Fenton-like reaction with different size ranges (30, 50, 300 nm) which could catalyse the catalytic oxidation of TMB similar to that of natural enzyme, HRP, as well as *o*-phenylenediamine (OPD) and diazoaminobenzene (DAB) in the presence of H_2_O_2_ at acidic pH conditions to yield different coloured products such as blue, orange, and brown, respectively [50]. The study revealed that the peroxidase-like activity of nanozymes was size-dependant where small-sized nanoparticles exhibited higher catalytic activity when compared to larger sized particles. Similar to natural HRP enzyme, the catalytic performance of iron oxide nanozymes also varies with pH and reaction temperature. However, unlike HRP, the Fe_3_O_4_ MNPs showed more robustness by remaining stable and retaining their catalytic efficiency at wide range of reaction temperatures (4–90 °C) and pH (1–12) [51]. Kinetic studies revealed that Fe_3_O_4_ MNPs have higher Michaelis–Menten constant (*K*_*m*_) value with H_2_O_2_ as the substrate in comparison with HRP (154 and 3.7 mM, respectively), suggesting the requirement of higher concentrations of H_2_O_2_ to achieve maximum catalytic activity, whereas Fe_3_O_4_ MNPs exhibit much lower *K*_*m*_ value with TMB as substrate than that of HRP (0.098 and 0.43 mM, respectively), showcasing higher affinity for TMB at same molar concentration [50]. Highly robust nanozymes with excellent intrinsic peroxidase-like activity, and low production cost make them suitable for a broad range of biosensing applications. Soon after Yan’s first report on the peroxidase-like activity of Fe_3_O_4_ MNPs [50], several reports have been published demonstrating nanozyme activity of iron oxide and other nanomaterials for biosensing applications. Wei and Wang developed a biosensing platform for the detection of H_2_O_2_ and glucose using peroxidase-mimicking Fe_3_O_4_ MNPs [52]. The mechanism behind the peroxidase-mimicking activity of Fe_3_O_4_ MNPs was based on Fenton-like reaction where the concentration of H_2_O_2_ played significant role in the substrate oxidation and generation of colorimetric signals (see Fig. 3). These initial findings encouraged rapidly growing interest for the applications of different iron oxide nanomaterials as peroxidase mimics with Fenton-type reactions for biosensing. Among them, magnetite nanoparticles have been studied extensively [51]. For example, Feng et al. demonstrated fabrication of ultrasensitive amperometric immunosensor for the detection of prostate specific antigen/PSA (with a lower detection limit of 0.13 pg mL^−1^) by exploiting Fenton-type reaction induced by Au/Fe-MOF with intrinsic peroxidase-mimicking activity [53]. Moreover, in recent years nanomaterials with single-atomic layer structures such as Fe nanozymes have shown outstanding intrinsic peroxidase-like activity based on Fenton-like reaction, which could be further utilized in different biosensing applications. For example, Du and co-workers proposed a new single-atom nanozyme (SAN) based on single Fe atoms anchor on N-doped carbons supported on carbon nanotube (CNT/FeNC) with brilliant peroxidase-like activity which means that it was able to catalyze H_2_O_2_ to generate hydroxyl radicals (·OH) though surface Fenton reaction between H_2_O_2_ and Fe ion. Further based on the superior peroxidase-like activity of CNT/FeNC and their ability to oxidize broad range of substrate oxidation, the single-atom nanozyme was utilized for different colorimetric biosensing applications [54]. Furthermore, there are several other iron-based nanomaterials such as iron chalcogenides (*e.g.* FeS nanoparticles, FeS nanosheets [55]), iron phosphates, doped ferrites (*e.g.* CoFe_2_O_4_ [56] and BiFeO_3_ [57]) have been reported for exhibiting peroxidase-like activities folioing the Fenton-type reaction mechanism (Fig. 3). Dai et al. reported micelle-assisted synthesis of Fe-S nanosheets with peroxidase-like activity [55]. The results revealed the higher peroxidase-mimicking activity of Fe-S nanosheets than of Fe-S spherical nanoparticle attributed due to larger surface area of nanosheets. In similar study, Fe-S nanoneedles have also shown improved peroxidase activity than spherical Fe-S nanoparticles [58].

Along with iron oxide nanomaterials, several other metal oxide nanoparticles have also been reported for exhibiting peroxidase-mimicking activities. A dual intrinsic enzyme-mimicking activity was observed for cubic cobalt oxide nanoparticles (Co_3_O_4_) [59]. Peroxidase activity of Co_3_O_4_ nanozyme was determined via catalytic oxidation of TMB where kinetic analysis revealed that nanozymes had higher affinity towards TMB but lower for H_2_O_2_ when compared to natural HRP. Further study also suggested that Co_3_O_4_ nanozymes were capable of transferring electrons between substrate and H_2_O_2_, instead of hydroxyl radicals, which lead to the catalytic oxidation of TMB. In another report, L-arginine-assisted hydrothermally synthesized gillyflower-shaped Co_3_O_4_ nanoparticles were tested for their intrinsic peroxidase-like activity [60]. Higher peroxidase-like activity was achieved due to their unique shape and larger surface area following with the similar mechanism as mentioned earlier in case of cubic Co_3_O_4_ nanoparticles. Vanadium pentoxide (V_2_O_5_) nanowires have also been reported to exhibit excellent peroxidase-like activity. In 2011, Thermal’s group reported that V_2_O_5_ nanowires are capable of mimicking natural vanadium haloperoxidase (V-HPOs) with a turnover frequency (*K*_*cat*_) of 2.5 × 10^3^ s^−1^ and *K*_*m*_ values for the oxidation of ABTS and H_2_O_2_ were found to be 0.4 and 2.9 µM, respectively, at pH 4.0 [61]. In another study, the same group have demonstrated that peroxidase-like activity of V_2_O_5_ nanowires enables to convert bromide into water and hypobromous acid (HOBr) in the presence of H_2_O_2_, this peroxidase mechanism was utilized as an alternative to traditional anti-biofouling agents to prevent marine-biofouling as it also produces singlet molecular oxygen during the reaction, which has strong antibacterial activity [62].

Due to the attractive electronic properties, biocompatible metal nanoparticles are of substantial significance for exhibiting peroxidase-like activity. However, it has been observed that several factors such as size, shape, surface charge, surface chemistry, pH, and reaction temperature play significant role in their catalytic behaviour [63]. Au NPs with positive or negative surface charges have been reported for their peroxidase activity [46, 64]. Cysteamine-modified Au NPs with positive surface charge showed better peroxidase-like activity when compared to citrate-capped negatively charge Au NPs [46]. It was also observed that the surface modification of Au NPs could interfere in their peroxidase-like activity. For example, bovine serum albumin (BSA)-coated fluorescent Au nanoclusters showed lower peroxidase-like activity than of naked Au NPs [65]. Similar to Au NPs, platinum nanoparticles (Pt NPs) were also reported for their peroxidase-like activity [66, 67]. Notably, their catalytic activity also depends on pH, temperature and size of the nanoparticles. Fan et al. have showed Pt nanoparticles with apo-ferritin (1–2 nm) exhibits high peroxidase-like activity with increasing pH and temperature [66]. Furthermore, cetyltrimethylammonium bromide (CTAB)-coated Pt nanocubes (10 nm) showed good peroxidase-mimicking activity at acidic conditions [67].

Carbon-based nanomaterials such as single-walled carbon nanotubes and graphene oxides were demonstrated to possess peroxidase-like activities similar to natural HRP. Qu and co-workers firstly established that carboxyl-modified graphene oxide (GO-COOH) exhibits intrinsic peroxidase-like activity, which can catalyse the TMB oxidation in the presence of H_2_O_2_ similar to HRP used in conventional immunosensing [68]. However, similar to other nanomaterials, the catalytic activity depends on size, pH, temperature, and H_2_O_2_ concentrations [68, 69]. For example, graphene quantum dots (GQDs) have showed better peroxidase-like activity when compared with larger graphene oxide sheets. The peroxidase-mimicking GQDs were further explored for different biosensing applications [70, 71]. These interesting findings have inspired to explore other carbon-based nanomaterials as peroxidase mimics such as carbon dots [72], carbon nitride [73], and Fe/N-doped carbon [74]. Several other bimetallic and MOFs have been reported to exhibit peroxidase-mimicking activities, thus showcasing the growing interest and recent efforts to develop biosensing applications based on novel nanozymes (Table 1).

### Oxidase-Mimicking Nanozymes

Oxidase enzymes are capable of oxidizing their substrate (*e.g.* amino acids, amines, alcohols) in the presence of molecular oxygen, which then converted into water (H_2_O) or H_2_O_2_. In contrast to peroxidase reaction, the catalytic reaction of oxidase enzymes does not require H_2_O_2_. Instead, they generate H_2_O_2_ and in few cases superoxide radicals. This in situ generation of H_2_O_2_ and superoxide radicals by oxidase leads to efficient oxidation of colourless substrate into its corresponding coloured products, thus making the enzyme a promising candidate for biosensing applications. In past few years, several nanomaterials have been reported to exhibit oxidase-mimicking activities. A schematic illustration of oxidase activity of oxidase-mimicking nanozymes is shown in Fig. 3.

As mentioned earlier, it is important to note that the catalytic behaviour of nanoparticles relies on the size, shape, composition, and surface chemistry of the nanoparticles. Altering these parameters can lead to either desired or unexpected nanozyme activities. For example, antioxidant ceria nanoparticles (CeNPs) have the ability to exhibit both catalase and SOD-like activity depending on the variations in Ce^+3^/Ce^+4^ ratio [75]; however, surface modifications of CeNPs with biocompatible dextran resulted in exhibiting oxidase-like activity [76, 77]. These studies from Perez’s group [76] demonstrated that the CeNPs with oxidase-mimicking activity could oxidize several colorimetric substrates (*e.g.* ABTS, 3,4-dihydroxyphenylalanine (DOPA) and TMB) under acidic pH conditions without the requirement of H_2_O_2_. The nanozyme activity was found to be highly depended on pH, size, and surface coating thickness of the CeNPs in agreement with the kinetic study the acidic pH, smaller size, and thinner surface coating favoured the oxidase-like activity with faster rate constant as compared to HRP (1 × 10^–7^ M^−1^ s^−1^ and 1 × 10^–8^ M^−1^ s^−1^, respectively). Later, the same group employed this oxidase mimicking CeNPs for the detection of tumour cells by conjugating poly acrylic acid (PAA) following with folic acid functionalization [77].

Along with CeNPs, few other metal oxide nanomaterials have also been explored exhibiting oxidase-like activities [78–82]. Cao and Wang reported the oxidase mimicking activity of Fe_2_O_3_ nanowires where they developed a glucose sensor by fabricating an array of Fe_2_O_3_ nanowires. The glucose sensing system showed a linear range of glucose detection (0.15–8 mM) with a limit of detection (LOD) of 6 mM [78]. In another report, Fe-Pt alloy was prepared using polyoxyethylene cholesteryl ether (a non-ionic surfactant) exhibiting intrinsic and robust oxidase-like activity with a tenfold increased reaction velocity as compared to other oxidizing nanomaterials reported [79]. Additionally, Deng et al. reported enhanced activity towards TMB substrate with chitosan-stabilized Pt NPs [31], which was enhanced by the presence of acid phosphatase (ACP), thus highlighting their potential as effective oxidase mimics for biomarker detection. Furthermore, copper-based nanomaterials have been explored widely as oxidase mimic. For instance, Cu_2_O/polypyrrole composites were reported to possess glucose oxidase (GOx) mimicking activity [81]. The study revealed the composites were capable of oxidizing glucose at basic pH conditions (0.5 M NaOH). Although the excellent oxidase-mimicking activity of Cu_2_O/polypyrrole composites leads to sensitive detection of glucose; however, there is still a need for further optimization of the physiological conditions of the reaction for a wide range of real-time applications [81]. In another study, Thermal and co-workers reported that sulphite oxidase (SuOx) mimicking molybdenum trioxide nanoparticles (MoO_3_ NPs) are capable of converting sulphite to sulphate under physiological in vivo conditions [82]. MoO_3_ NPs (with an average diameter of 2 nm) were functionalized with dopamine to link triphenylphosphonium (TPP) ligands for targeting the mitochondrial through the membrane barrier. The kinetic study revealed that the *K*_*m*_ value of MoO_3_-TPP nanoparticles for SO_3_^2−^ oxidation was found to be 0.59 ± 0.02 mM and was equivalent to those of goat SuOx and human SuOx mutant R160Q [82].

Citrate-capped Au NPs have been broadly investigated for their wide range of biomedical applications. Among them, catalytic activities of “naked” or citrate-capped Au NPs have attracted great interest owing to their potential for biosensing applications. It is also evident that surface coating with different molecules could lead to suppression or enhancement of the enzyme-mimicking catalytic activities of the citrate-capped Au NPs (as discussed earlier). Rossi and co-workers showed that the citrate-capped Au NPs exhibiting excellent GOx mimicking activity by catalysing the oxidation of glucose in the presence of dissolved oxygen [83]. However, this report was surprising as other metal nanoparticles such as Ag, Pt, Cu, and Pd did not possess any significant oxidase-like activity [41, 83]. Further mechanism studies revealed that the oxidase activity of citrate-capped Au NPs depends on the hypothesis of Eley–Rideal mechanism of catalysis in which glucose was adsorbed on the Au NPs surface followed by the oxidation by molecular oxygen and forming gluconic acid and H_2_O_2_ as products [83]. However, the kinetic study revealed that the nanozymes was ~ 55 times less active than that of native enzyme [83, 84]. Another study performed by Fan and his group was to demonstrate an innovative microRNA sensing technology by utilizing the oxidase mimicking activity of Au NPs [85]. In their study, they have utilized the different affinities of nucleic acids such as ssDNA, dsDNA towards Au NPs, which facilitate the surface tuning and growth of the nanoparticles followed by coupling of the system with natural enzyme (HRP) to generate chemiluminescent and colorimetric signals for specific detection of single-base-pair mismatches [85]. Several other nanomaterials possessing oxidase-like activity and their applications in biosensing are listed in Table 1.

### Catalase-Mimicking Nanozymes

Natural catalase enzymes are of considerable significance as they are able to catalyse the cellular H_2_O_2_ decomposition into water and molecular oxygen. The dismutation of superoxide radical by natural SOD enzymes present in the cell attributes to the generation of excess cellular H_2_O_2_. Additionally, H_2_O_2_ being a stable and less active molecule, it can play a dual role in biological systems as a signalling agent and as a non-radical reactive oxygen species [86]. It is important to note that an excess amount of H_2_O_2_ can lead to the rise of several diseases if not controlled. Thus, it is important to remove excess amount of H_2_O_2_ in the cytoplasm by converting them into water and molecular oxygen using catalase enzymes. In the past few years, researchers have found many metal and metal oxide-based nanomaterials exhibiting catalase enzyme-like activities including Au, Ag, Pd, Pt [87, 88], Au@Pt [89], cerium oxide [90], iron oxides [91], and cobalt oxide nanoparticles [92, 93]. A schematic representation of catalase-mimicking activity of nanozymes is shown in Fig. 3.

Interestingly, most of these reported nanomaterials possessed catalase-like activity along with other enzyme-mimicking activities. Furthermore, coexistence of these switchable enzyme-mimicking characteristics majorly depends on the pH and temperature of the catalytic reaction. For example, under basic pH conditions metal nanoparticles act as catalase mimics where H_2_O_2_ would favour the acid-like decomposition into H_2_O and O_2_ on the metal nanoparticles surface [87, 94]. On the other hand, under acidic pH conditions nanozymes favoured the peroxidase-like activity [94]. Furthermore, Pt and Pd nanoparticles demonstrated higher catalase-mimicking activities than that of Au and Ag NPs [87]. Several advantages of catalase-mimicking Pt nanozymes allow them to be exploited for various photodynamic therapy (PDT) and biosensing applications. For example, Au core/Pt shell nanoparticles (Au@Pt NPs) have been applied to attenuate tumour hypoxia and enhance immune cell-mediated cytotoxicity [89]. Similarly, depending on the pH conditions few other nanomaterials such as Co_3_O_4_, zirconium dioxide (ZrO_2_), and Prussian blue (PB) also showed higher catalase-mimicking activity at higher pH (basic) conditions [92, 95]. Mu and co-workers found a dual enzyme-mimicking activity of Co_3_O_4_ nanoparticles, where nanozymes were capable of showing weak catalase-like activity along with peroxidase-mimicking properties [96]. In the same study, they have further demonstrated that, by changing the pH from acidic to neutral or basic conditions, the catalase-like activity of Co_3_O_4_ nanozymes was enhanced significantly, hence proving the pH dependent existence of dual enzyme-mimicking phenomenon. Later, the same group (Mu and co-workers) demonstrated electrocatalytic sensing of hydrogen peroxide using catalase-mimicking Co_3_O_4_ nanoparticles. Further mechanism studies revealed that, firstly, H_2_O_2_ absorbed on the surface of Co (II) would decompose into ·OH following with the formation of OOH^¯^ via the reaction between H_2_O_2_ and OH^¯^ and then ·O_2_H would generate after the interaction between OOH^¯^ and Co (III) [92]. Finally, H_2_O and O_2_ would be produced via the reaction between two radicals. In another similar study, Prussian blue with multiple redox forms showed excellent switchable catalase-like activities [95]. The results demonstrated that at higher pH conditions, due to the low redox potential of H_2_O_2_/O_2_, it could easily oxidize and reduce PB to BG/PY (Berlin green/Prussian yellow) and vice versa, respectively, along with the production of O_2_.

In another study, Singh and Singh [97] demonstrated the dual enzyme-mimicking characteristics of CeNPs as SOD and catalase mimics. The study revealed that in biological buffered solutions the catalase mimetic CeNPs (with high oxidation state Ce^+4^/^+3^) are more robust and stable than that of SOD mimetic CeNPs (with high oxidation state Ce^+4^/^+3^) without losing their catalytic activity [97]. Inspired from the interesting findings on the stability of catalase mimetic CeNPs, the same group further utilized CeNPs nanozymes for the protection of hepatic cells from the H_2_O_2_ induced cytotoxic and genetic damaged at cellular level [98]. In their study, human hepatic cells were exposed to 3-aminotriazole (3-AT) which artificially inhibit the cellular catalase enzyme activity which leads to the elevation in the cellular concentration of H_2_O_2_. The results showed that the degradation of H_2_O_2_ was carried out by the catalase-mimicking CeNPs without elicitation of the natural antioxidant defence system. These results demonstrated the suitability of catalase-mimicking CeNPs as a pharmacological agent in case of impaired natural catalase enzyme dysfunctionality in vivo conditions [98]. Iron oxide nanoparticles were also reported to possess catalase-like activity; however, there is a lack of supporting evidence as most of the extensive studies involve iron oxide nanozymes as peroxidase mimics. Chen et al. [94] have reported pH dependent low catalase-like activity of iron oxide nanoparticles at neutral pH (7.4). Their study also revealed that at acidic pH iron oxide nanozymes act as peroxidase mimic and generation of hydroxyl radicals occurred due to the “Fenton-like chemistry” [94] (Fig. 3). Motivated from these findings, other nanomaterials with peroxidase-mimicking activity could also be investigated to determine the switchability of enzyme-mimicking activities such as peroxidase and catalase based on the pH variations. In addition, the further molecular mechanism study involved in these interesting phenomena would lead to broaden the area of biosensing and biomedicine applications in near future. Nanozymes possessing catalase-like activity and their applications in biosensing are also summarized in Table 1.

### Superoxide Dismutase (SOD)-Mimicking Nanozymes

Natural SOD enzymes play a crucial role in the context of mammalian cellular protection as they are able to eliminate superoxide anions O_2_^·¯^ (one of the reactive oxygen species, (ROS)) by producing H_2_O_2_ and O_2_ via dismutation reaction of O_2_^·¯^. Impaired dysregulation of the ROS generation at cellular level could lead to elevated oxidative stress and adverse effects to the living body. Due to the lack of stability and high production cost of natural SOD enzymes [32], several efforts have been given in the past few years to develop an effective alternative as a SOD mimics. In one such effort, researchers were able to develop manganese-consisting biscyclohexylpyridine complex (M40403) exhibiting SOD-like activity which had a significant impact on the cellular protection from superoxide radicals but their activity was restricted towards certain selective in vivo applications [99]. For example, M40403 did not show any protective effect against cisplatin-induced hair cell loss in cochlear cultures, whereas it showed its protective effects against gentamycin treated cell lines [100]. Motivated by this work, several nanomaterials have been reported to exhibit SOD-mimicking activities [101–106]. A schematic representation of the SOD-mimicking activity by nanozymes is shown in Fig. 3.

Among the SOD-mimicking nanozymes, ceria nanoparticles (CeNPs) are most extensively studied. Self group first reported the SOD-mimicking activity of CeNPs with better catalytic efficiency [86, 107, 108]. Further kinetic study revealed that CeNPs showed higher SOD-mimicking activity in comparison with native CuZn SOD with rate constant of 3.6 × 10^9^ M^−1^ s^−1^ and 1.1 × 10^5^ M^−1^ s^−1^, respectively [109]. Although a detailed mechanism behind the superoxide anion scavenging ability of CeNPs needs to be verified, several studies have reported that the SOD-mimicking activity of CeNPs mostly attributed to the presence of electron shuttle between their mixed oxidation states (Ce^3+^ and Ce^4+^) [110]. Additionally, the higher ratio of Ce^3+^/Ce^4+^ of the surface “Ce” atoms resulted in SOD mimetic activity, whereas lower ratio leads to catalase-like activity hence, proving the coexistence of dual enzyme-mimicking activities, as mentioned in earlier sections [110]. In view of the connotation between oxygen vacancy and Ce^3+^, more surface oxygen vacancy could be generated by reducing the size of CeNPs resulting in high Ce^3+^ [109, 111]. Therefore, CeNPs with size range less than 5 nm were investigated extensively for their SOD-mimicking activities. Furthermore, enhanced SOD-mimicking activity of CeNPs could be achieved by doping them with Zr/La (lanthanum) atoms to generate more oxygen vacancies [112, 113].

Although most of the studies about nanozymes exhibiting SOD-like activity have focused on CeNPs, few other metal, metal composites and carbon-based materials were also reported to exhibit SOD-like activity. He et al. demonstrated ·OH as the ROS induced by Au NPs [101]. In the AuNP-catalysed decomposition of H_2_O_2_, generation of hydroxyl radicals and oxygen were strongly dependent on pH. Lower pH was required for the generation of hydroxyl radicals, while a higher pH was required for the generation of oxygen. Thus, Au NPs demonstrated dual enzyme characteristics, as both SOD and catalase mimetics, which could be tuned by adjusting the pH [101]. Apo-ferritin encapsulated Pt nanoparticles were reported to exhibit SOD-like activities [114, 115]. Although the Pt nanoparticles could retain their SOD-mimicking activity in cell culture (i*n vitro*) studies, the overall efficacy of SOD mimetic was significantly lower than that of CeNPs. This makes them suitable for applications in the context of cellular protections in the field of biomedicine. However, many of these findings could not effectively elucidate the exact mechanism and determining factors behind the type of activity (either catalase or SOD) showed by CeNPs, which could possibly be because of insufficient characterizations.

Fullerenes (C60) and their derivative carbon-based materials have also been employed to scavenge free radicals and super oxide anions to protect neuron cells from oxidative damage owing to their SOD-mimicking activity [116, 117]. Further electron paramagnetic resonance (EPR) studies suggested that fullerenes could scavenge superoxide anions, as well as hydroxyl radicals efficiently [118, 119]. In further studies, Dugan’s group also reported a tris-malonic acid derivative of fullerene with a 100-fold lower SOD-mimicking efficiency, in comparison with natural SOD enzymes [118].

Application of nanozymes has gained much popularity in recent years due to their excellent intrinsic enzyme-mimicking properties and stable nature in comparison with natural enzymes. Owing to the intrinsic physiochemical and optoelectronic properties of nanoparticles depending on their size, shape and even compositions, nanozymes could be able to work amenably based on the working environment and being approachable to numerous biomedical settings. There is an unambiguous consensus that the enzyme-mimicking activities of nanomaterials depend on several factors such as pH, level of oxygenations, temperature, and redox conditions. By altering these reaction conditions, the catalytic properties of nanozymes could be regulated. Various nanomaterials were reported to exhibit multiple enzyme-mimicking activities (mostly dual enzyme-mimetic properties) by pH variations, or change in the oxidation states of the nanoparticles, the desired nanozymes activity could be achieved. These interesting findings provide the opportunity to utilize these nanozymes in several biosensing applications. Further, applications of these nanozymes in POC-based biosensors following ASSURED criteria would be discussed in the following sections.

## Nanozymes for the Development of Biosensors and POC Devices Following ASSURED Criteria

Over the past few decades, the development of low-cost, sensitive, and deliverable detection techniques has become an emerging trend in the medical and healthcare industry. Among the emerging detection techniques, point-of-care (POC) for diagnosis and detection has been considered as a definitive goal in biosensor and bioassay development. Enzyme-mimetic nanomaterials possess several advantages compared with natural enzymes, such as excellent robustness with high stability under harsh physiological conditions, cost effectiveness, long-term storage, ease of large-scale production and size-, shape-, composition-dependent catalytic activities [183]. Furthermore, the nanozymes display unique properties such as availability of large surface-to-volume ratio, making them suitable for various surface modifications and bioconjugations. In addition, nanozymes’ smart response to peripheral inducements, capability of self-assembly along with the enzyme-mimicking activities enable their suitability for a wide range of diversified applications, such as *in vitro* sensing (detection of H_2_O_2_, glucose, nucleic acid, proteins biomarkers for cancer, ion *etc.*), *in vivo* sensing, bio-imaging, tissue engineering, therapeutics and beyond [3, 39, 183]. However, amongst these applications, recent progress in nanozymes’ applications in biosensing is remarkable due to their signal transducing capabilities depending on their size, shape, and properties such as fluorescence, biocompatibility, and magnetism, electrical and thermal conductivity. Considering these characteristics suitable for biosensor development, POC bioassays would be one of the most favourable applications of enzyme-mimetic nanomaterials [3, 184]. Therefore, enzyme-mimicking nanozyme-based POC devices have been developed recently for the ultrasensitive and rapid detection of analytes such as biomarkers, contaminants, pathogens and toxins [185]. However, the requirement of specialized equipment and complicated operations of these nanozyme-based POC bioassay systems limit their applications in on-site remote locations with limited resources. To overcome these drawbacks, there has been a drive to develop nanozyme-based POC devices following the guidelines of ASSURED criteria (Fig. 1) implemented by the World Health Organization (WHO) [186]. Recent developments in the field of POC devices with suitable nanozyme as a key component in sensing mechanism following the ASSURED criteria will be further discussed in the following sections.

### Affordability of Nanozymes-Based Biosensors

**Fig. 4:**
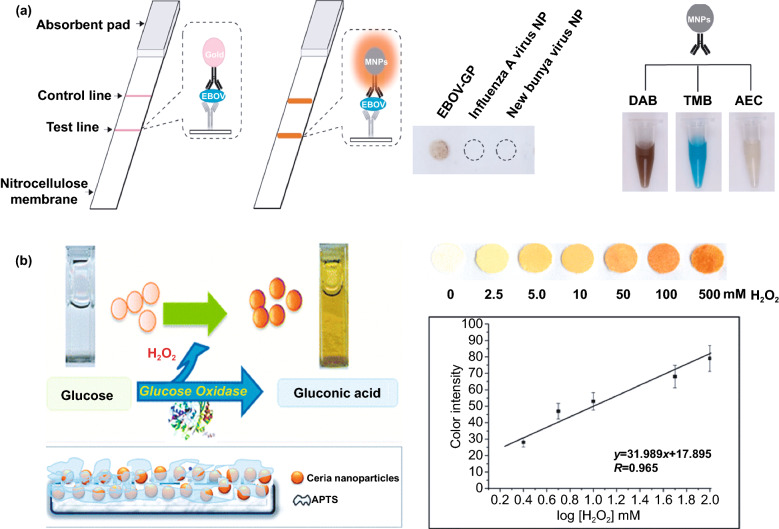
**a** Nanozyme-strip designed for the detection of Ebola virus. It shows standard colloidal gold strip, MNPs as nanozyme probe, which amplifies the signal by generating colour reaction and can be visualized by naked-eye. Specificity of the nanozyme probe towards EBOV-GP, but not for other virus proteins such as nucleoprotein of influenza A virus or New Bunya virus and the peroxidase-mimicking activity of antibody coated nanozymes towards different peroxidase substrates such as DAB, TMB, AEC [121]. Copyright 2015 Elsevier. **b** Paper-based bioassay for the detection of glucose using ceria NPs as nanozyme. Schematic representation of ceria nanoparticle-immobilized paper in APTS, colorimetric response of ceria-immobilized paper strips depending on the different range of H_2_O_2_ concentrations and linear calibration curve for H_2_O_2_ detection using ceria-immobilized paper strips [190]. Copyright 2011 American Chemical Society

One of the major drawbacks of conventional biosensing systems for field-level applications is the involvement of expensive instrumentation with complex handling nature, or expensive cost of detection, which makes them not feasible and affordable for resource-limited areas. To fall into the ASSURED criteria set up by WHO, affordability has been considered as one of the key components for the fabrication of nanozyme-based POC devices. POC devices for early diagnosis of infectious diseases, biomarkers, toxins, pathogens, *etc*., have to be rapid, robust and sensitive, but low in cost and economically sustainable for on-site applications. Considering these criteria in the past few years, the paper-based microfluidics, lateral flow assay (LFA) or lateral flow stripes (LFS)-based colorimetric and electrochemical biosensors have emerged as most promising POC devices for detection and quantification of analytes from a different specimen at field-level application. The utilization of paper as a base material for the biosensing platform design reduces fabrication cost significantly. It is well known that the sensory mechanism of paper-based bioassay relies on the three basic key components: (i) conjugation of sample analyte, (ii) detection, and (iii) signal amplification. Different metal and metal-oxide nanoparticles with high enzyme-mimetic activity have been employed as detection and signal amplification probes in low-cost POC devices for higher sensitivity [37, 187]. However, apart from the low-cost fabrication process of affordable POC biosensors, several aspects of nanozymes also play a significant role in enabling low-cost detection [3, 184]. Firstly, as mentioned earlier in section 2.0, one of the main advantages of using nanozymes is facile synthesis without the involvement of expensive chemicals and sophisticated instrumentations, which indirectly reduces the overall fabrication cost. This greatly enables the applications of numerous metals, metal oxides, and MOFs nanozymes for affordable biosensor developments. As an illustration, Duan and co-workers prepared Fe_3_O_4_ nanozyme-based immunochromatographic strips (ICS) capable of detecting glycoprotein (GP) of the Ebola virus (EBOV–GP) at 1 ng mL^-1^ with higher sensitivity in comparison with a standard colloidal gold strip [121] (Fig. 4a). As discussed in the earlier sections, Fe_3_O_4_ magnetic nanoparticles were reported as the first nanozyme with peroxidase-like activity and the facile synthesis could be done using low-cost precursors [50]. Likewise, in their study, the Fe_3_O_4_ nanozyme was prepared by simple hydrothermal method where FeCl_3_ precursor salt mixed with ethylene glycol and sodium acetate (NaAc) was kept in sealed autoclave for 16 h followed by rinsing and drying at 50 ℃. Furthermore, the Fe_3_O_4_ nanozyme conjugated with antibody was prepared using simple EDC/NHS (1-ethyl-3-(3-dimethylaminopropyl)carbodiimide/N-Hydroxysuccinimide) carbodiimide cross-linking method. One can argue on the fact that the involvement of antibody conjugation process could increase the cost of the biosensor fabrication. However, when it is compared with conventional ELISA using HRP-conjugated antibody as a colorimetric signal amplifier that requires additional step, the cost of fabrication clearly increases so as the complexity [121]. Besides, due to higher intrinsic peroxidase-like activity of Fe_3_O_4_ nanozymes towards its substrate 3,3'-diaminobenzidine (DAB), the signal amplification was enhanced by 100-fold in comparison with conventional ELISA [121]. In another similar study, a simple and low-cost photoelectrochemical (PEC) immunoassay was developed by Li et al. [141]. Microwave-assisted synthesis of histidine-modified Fe_3_O_4_ nanozymes was demonstrated following with the antibody (specific to prostate-specific antigen (PSA)) conjugations similar to the above-mentioned procedure. The utilization of Fe_3_O_4_ nanozymes with low production cost as a substitute of natural enzymes (*e.g.* HRP; widely used in conventional immunoassays) enables the cost reduction for biosensor fabrication. Similar to low-cost nanozyme synthesis, reusability of nanozyme-based biosensors and POC devices is another point of concern towards affordable POC applications as it reduces the cost per test by reusing the same set-up. There are many reports established regarding the fabrication of inexpensive, reusable paper-based bioassays for disease biomarker detection with enzyme-mimetic nanoparticles [188–190]. For example, Maryan Ornatrska and co-workers reported the fabrication of cerium oxide (CeO_2_)-based bioactive sensing paper strip for the detection of H_2_O_2_ and glucose (Fig. 4b). The paper-based assay with 4.3% reproducibility could be used for at least 10 constructive measurements cycle without losing its activity. Hence, this reduces the cost involved in each experimental cycle while retaining its efficiency. The functionalization of CeO_2_ nanoparticles in the bioactive paper was performed by simple electrostatic adsorption method (Fig. 4b). In the presence of higher concentration of H_2_O_2_ produced by oxidation of glucose, the physicochemical properties of CeO_2_ nanoparticles changes due to the changes in oxidation state and formation of surface complexes resulting in colorimetric detection of glucose [190] (Fig. 4b). In addition to reusability of nanozyme-based biosensors, the other advantages of nanozymes such as high thermal stability and mild preservation conditions could play an essential role in reducing the cost of the biosensor fabrication. One good example of fabrication of low-cost biosensor utilizing highly stable nanozymes was established by Tran et al. [191]. In their work, they demonstrated fabrication of enzymatic biosensor for the detection of glucose and H_2_O_2_ utilizing nanocomposite containing FeOOH and N-doped carbon nanosheets. The Fe-CN nanocomposites with intrinsic peroxidase-like activity showed excellent stability up to 90 days without compromising its catalytic efficiency. This highly stable nature of the nanozyme ultimately helped in reducing the fabrication cost. From these findings, it can be observed that the applications of nanozymes have the potential to not only increase the sensitivity of the detection in comparison with natural enzymes but also to reduce the cost of the fabrication process significantly. However, it is noteworthy to mention that despite cost-effectiveness and facile synthesis of nanozymes, simple but effective surface modification strategies with several specific ligands (or biorecognition molecules) to enhance the sensing specificity without losing their catalytic properties remain one of the most significant concern for the development of efficient and affordable POC devices.

**Fig. 5:**
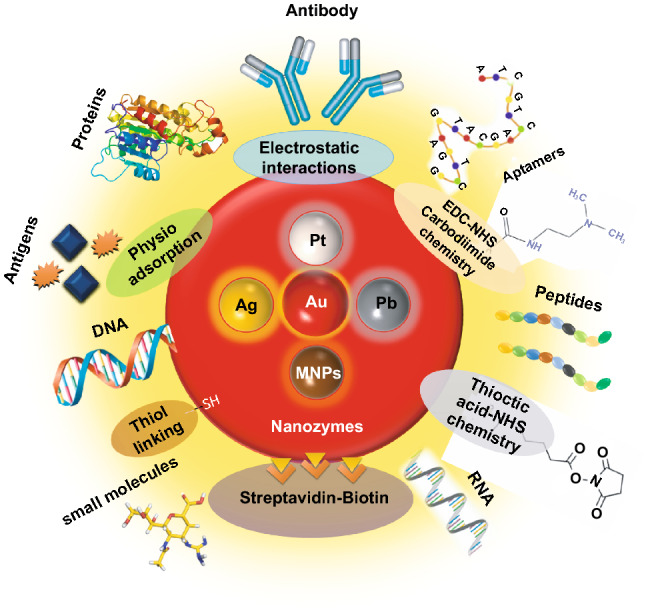
Different simple and effective conjugation chemistry for the surface modification of nanozymes with numerous biorecognition ligands

**Fig. 6:**
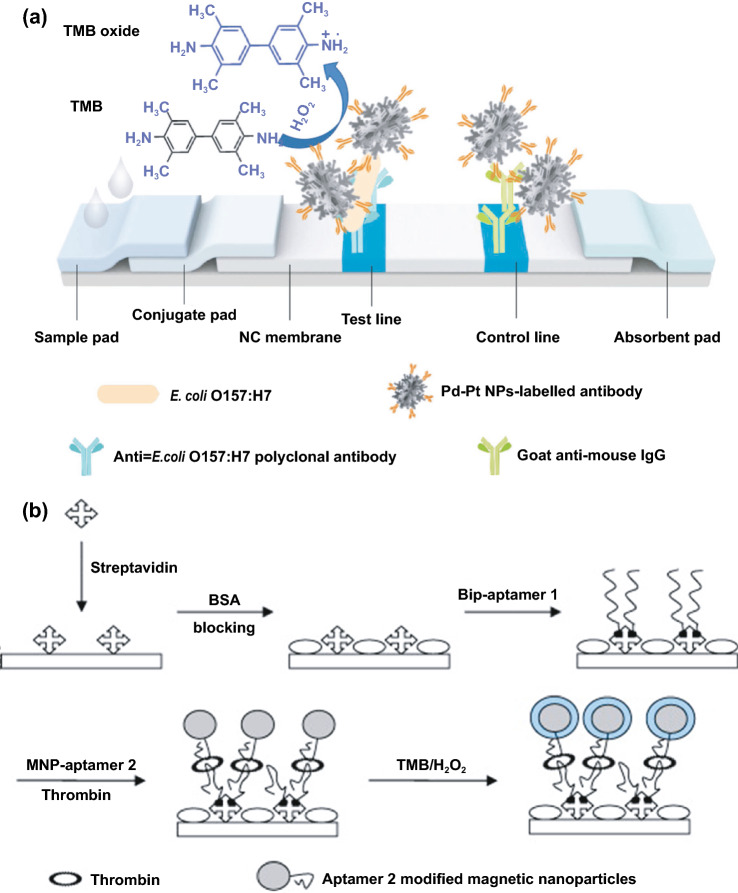
**a** Schematic illustration of Pd–Pt nanozyme-based lateral flow assay for the detection of *Escherichia coli* O157:H7. TMB = 3,3′,5,5′-tetramethylbenzidine; NC = nitrocellulose; NP = nanoparticles [133]. Copyright 2018 Elsevier. **c** Schematic illustration of the strategy behind the Fe_3_O_4_ MNPs linked colorimetric aptasensors assay for the detection of thrombin [192]. Copyright 2010 Elsevier

Interestingly, excellent biocompatibility of nanozymes along with ease of surface tunability with numerous desired biorecognition ligands through simple and widely accessible conjugation chemistry compared to complex organic counterparts have reduced the cost of biosensors fabrication and made it more approachable. Several biorecognition elements, such as antibody [130, 133], aptamers [174, 192], antigens [122], chemical linkers [193]*,* have been conjugated with nanozymes using simple conjugation chemistry for low-cost biosensor developments (Fig. 5). Han et al. have developed a nanozyme-based lateral flow assay for *Escherichia coli* O157: H7 detection with a higher sensitivity of 9.0 × 10^2^ CFU mL^-1^ in milk sample (Fig. 6a) [133]. The LFA was fabricated based on the sandwich immunoassay principle, where spherical palladium–platinum (Pd–Pt) nanozyme modified with an anti *Escherichia coli* O157: H7 monoclonal antibody (mAb) was used as a detection probe. The nanozyme@mAb conjugates were prepared based on simple physical interaction between Pd–Pt and mAb at basic pH (8.2-8.5) conditions where ionic interactions between negatively charged Pd–Pt and positively charged mAb resulted in conjugate formation. This simple conjugation technique without the involvement of costly reagents and organic linking counterparts resulted in cost reduction of the LFA fabrication process. In contrast, for conventional immunoassay, preparation of natural enzyme-antibody conjugates required costly reagents and specified organic linkers to conjugate the antibody with the enzyme without affecting the active sites, and thus, making the process more complex and costly due to their less flexibility towards the conjugation process [194]. The signal amplification for the detection of *Escherichia coli* O157: H7 was based on the excellent peroxidase-like activity of Pd–Pt nanoparticles, which catalysed the oxidation of TMB in the presence of H_2_O_2_ [133]. In another similar study, Cheng and co-workers demonstrated the application of mesoporous core–shell palladium@platinum (Pd@Pt) nanoparticles as a signal amplifier in dual lateral flow immunoassay (LFIA) integrated with a smartphone device for detection of *Salmonella enteritidis* and *Escherichia coli* O157: H7 [130]. They also have utilized the similar conjugation chemistry based on physisorption of mouse anti-*S. enteritidis* monoclonal antibody (1 mg mL^-1^) and mouse anti-*E. coli* O157:H7 monoclonal antibody (1 mg mL^-1^) on the surface of Pd@Pt nanozymes. The elevated peroxidase-like activity of Pd@Pt nanozyme resulted in excellent sensitivity of the dual LFIA device towards target pathogens with estimated recoveries ranging from 91.44–117.0%, respectively [130]. Similar to antibodies, aptamers (oligonucleotide/peptide molecules) are also one of the widely explored target specific detection ligands, which have been employed for the developments of biosensors. Due to easy acquisition with short preparation and lower cost, aptamers could be purchased directly from the manufacturing companies [195, 196]. Besides, use of different straightforward cross-linking chemistries using simple chemical linkers or utilization of modified aptamers for easy conjugations could result in lowering the overall cost of biosensor fabrication. Thus, it is believed that aptamers used in nanozyme-based biosensors have the potential to be a suitable candidate in terms of affordability following ASSURED criteria. To give an example, Yang and his group have demonstrated the development of Fe_3_O_4_ nanoparticles linked colorimetric aptasensors for the detection of thrombin with a LOD of 1 nM. The chitosan-modified Fe_3_O_4_ nanozymes were prepared through one-pot solvothermal method using low-cost precursor FeCl_3_, after that nanozymes were conjugated with amino-modified aptamers (15 monomeric units) under simple shaking conditions using glutaraldehyde as amino linker (Fig. 6b).The excellent intrinsic peroxidase-mimicking activity of Fe_3_O_4_ nanozymes retained after the conjugation and resulted in colorimetric signal generation at 652 nm in the presence of thrombin (in the range between 1 and 100 nM) *via* TMB oxidation [192]. The finding shows that the conjugation between aptamers and nanozymes could be achieved through simple and elementary pathway resulting in total cost reduction of the fabrication process and could be considered as affordable biosensing platforms for future applications.

**Fig. 7:**
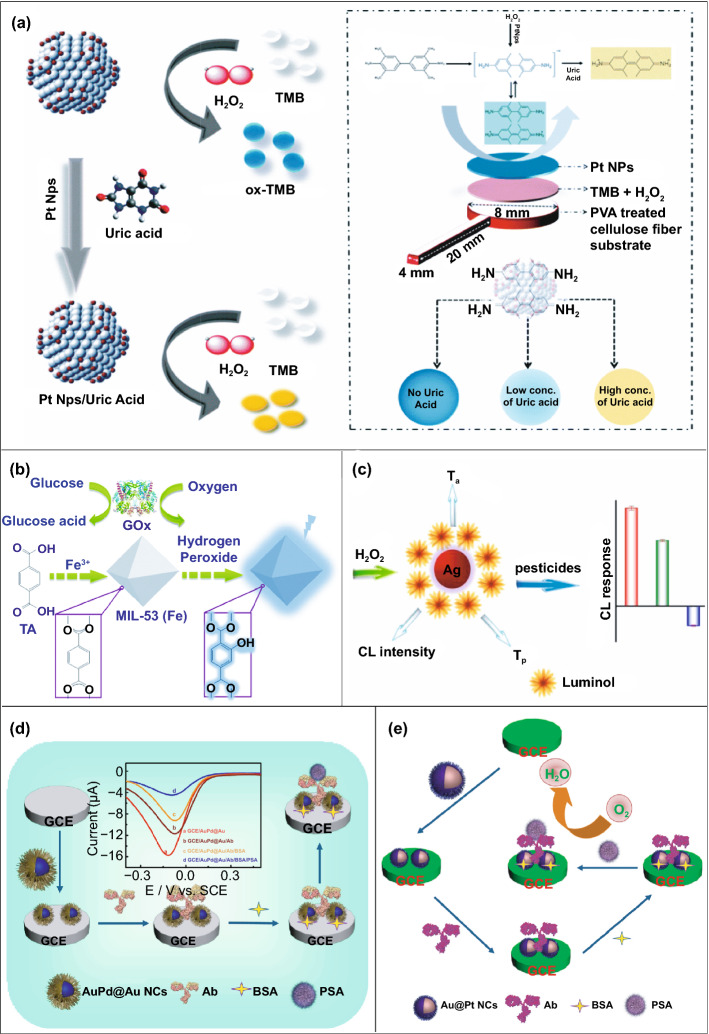
**a** Representation of the UA detection using PtNPs. It shows the preparation of cellulose strip consisting PtNPs and colour changes after addition of different concentration of UA [126]. Copyright 2019 Royal Society of Chemistry. **b** Principle behind the fluorescent-based detection of glucose using MIL-53(Fe) bifunctional nanozyme [200].Copyright 2018 Royal Society of Chemistry. **c** Schematic illustration of the principle of the CL sensor array based on the triple-channel properties of the luminol-functionalized Ag nanoparticles and H_2_O_2_ chemiluminescent (Lum AgNPs − H_2_O_2_ CL) system utilized for the detection of carbamate pesticides and organophosphorus [201]. Copyright 2015 American Chemical Society. **d** Schematic representation of the fabrication of an immunosensor based on Au@Pd@Au nanocluster nanozymes for the detection of PSA. Inset shows the differential pulse voltammetry (DPV) curve of different nanocomposites [204]. Copyright 2018 Elsevier. **e** Construction of an electrochemical immunosensor based on Au@Pt nanocluster nanozymes for the detection of PSA [203]. Copyright 2018 Elsevier

Along with the low cost of synthesis and ease of accessible conjugations, nanozymes can also catalyse for a range of different substrates, to generate different signals (colorimetric, fluorescent, chemiluminescent, or electrical signal). Therefore, this can allow flexibility in design and development of POC device in favour of transducer simplification and cost of fabrication. Nanozyme-based colorimetric biosensors have attracted great attention owing to their ability to catalyse different colorimetric substrates, such as TMB, o-phenylenediamine (OPD) and 2,2’-azino-bis(3-ethylbenzothiazoline-6-sulphonic acid) diammonium salt (ABTS) to produce colorimetric signals for a low-cost detection of wide range of analytes based on the colour variations [131, 197–199]. For example, Muhsin Ali and co-workers have developed citrate-capped-platinum nanoparticles (PtNPs)-based cellulose paper strip (Fig. 7a) for sensitive detection of uric acid (UA) with a linear response from 0–8 mM. The intrinsic peroxidase-like activity of nanozymes to oxidize TMB as a colorimetric substrate was employed for the colorimetric detection. The reaction colour changes from colourless to dark blue in the absence of UA, and then colour changes from dark blue to yellow after addition of UA due to the further oxidation of TMB. Naked-eye visualization of colorimetric signals produced by enzyme-mimicking activity of Pt nanozymes made the biosensing mechanism simple, cost effective, and efficient without the involvement of sophisticated instrumentation [126]. In another similar example, Pt@Au bimetallic nanozymes exhibiting peroxidase-mimicking activity were used for the detection of malathion [145]. The bimetallic nanozymes were capable of catalysing the oxidation of o-phenylenediamine (OPD) (chromogenic substrate) in the presence of H_2_O_2_ to generate a colorimetric signal. These findings provide evidence of flexibility of nanozymes in terms of choosing a substrate for low-cost colorimetric biosensor development. In addition to colorimetric biosensing, the ability of nanozymes to catalyse fluorogenic molecules leads to the development of low-cost nanozyme-based fluorescence biosensors. For instance, Lin and co-workers synthesized MIL-53(Fe) nanozymes with enzyme-mimicking activity similar to HRP and capable of catalysing the oxidation of terephthalic acid (TA), a fluorescent probe for hydroxyl radicals (Fig. 7b) [200]. Further working mechanism studies suggested that, nanozyme-assisted oxidation of TA lead to the production of 2-hydroxyterephthalic acid (TAOH) (a fluorescent product) which is being facilitated by the presence of H_2_O_2_, generated by the hydrolysis of glucose in the presence of GOx [200]. They have successfully employed the phenomenon to develop a highly sensitive fluorescence biosensor for the detection of glucose with a LOD of 8.44 nM. Unique optical and electrical properties of metal nanoparticles along with excellent enzyme-mimicking abilities have endowed the platform for the development of low-cost biosensors with chemiluminescence and electrochemical signals as transducing elements. He et al. [201] fabricated a simple facile chemiluminescent biosensor for the sensitive detection of carbamate and organophosphorus pesticides based on luminol-functionalized Ag nanoparticles (Lum-AgNPs) and H_2_O_2_ chemiluminescent system (Fig. 7c). Based on these findings, the same group developed a chemiluminescent biosensor for the detection of organophosphorus pesticides using Au-immobilized iron-based metal organic gels (MOGs) (Au NPs/MOGs), exhibiting intrinsic peroxidase-mimicking activity along with outstanding chemiluminescent properties in the presence of H_2_O_2_. Au NPs/MOGs nanozymes with decreased Fermi energy could facilitate the electron transfer between luminol and H_2_O_2_, resulting in H_2_O_2_ decomposition and oxidation of luminol compounds which leads to the emission of enhanced chemiluminescent signals for further analysis [202]. It is interesting to note that the flexible nature of catalytic properties of nanozyme-based biosensing system could lead to multiple possibilities for acquiring the qualitative and quantitative measurements of the amplified signals in the form of fluorescence, colorimetric, chemiluminescent, consequently making the fabrication process more flexible and affordable in terms of cost involvement. Despite the fact that numerous nanomaterials have been created for imitating various natural enzymes, redox enzyme mimics, particularly peroxidase mimics, remain dominating for biosensing applications. Other than peroxidase, relatively few examples of biosensors employing enzyme-mimicking phenomena have been reported earlier. Given the diversity of catalytic properties of nanozymes more efforts should be focused on creating innovative techniques for building nanozymes-based biosensing strategies. Consequently, nanozyme-based biosensor development would not only be more flexible, but also more affordable to detect a broader range of analytes. This might be accomplished by exploiting sensing signals produced during catalytic reactions, e.g. electrochemical signals.

Several electrochemical biosensors have been developed in recent years using the electrical signals produced by nanozyme-assisted catalytic reactions [203–205]. Wang and co-workers reported the fabrication of label-free electrochemical immunosensors utilizing dendritic core–shell AuPd@Au [204] and Au@Pt [203] nanozymes for the detection of prostate-specific antigens (PSA). The former electrochemical immunosensors were constructed based on peak currents generated by the transfer of electrons from a probe to an electrode, facilitated by H_2_O_2_ reduction catalysed by AuPd@Au nanocrystals (Fig. 7d). Increased concentration of PSA in the samples led to the formation of immunocomplex, which resulted in the suppression of peak currents by blocking the electron transfer in the electrode. This resulted in a sensitive detection of PSA with a LOD of 0.078 ng mL^-1^. The later was dependent on the electrical signals (peak currents) produced by the catalytic activity of Au@Pt towards oxygen reduction reaction (ORR), which resulted in lowering the detection limit of PSA up to 0.018 ng mL^-1^ (Fig. 7e) [203, 204].

Based on all the interesting findings mentioned above, it can be observed that the facile and low-cost synthesis of nanozymes, ease of surface conjugation with numerous ligands based on the specificity of the target analyte, reusability, recyclability, and the flexibility to choose the transducing element depending upon the substrate specific catalytic reactions, have played an immense role in reducing the fabrication cost of biosensors, making them more economically sustainable. Furthermore, the other benefits of nanozymes over natural enzymes, such as moderate preservation conditions and high thermal stability, might play a significant role in lowering the cost of the biosensor production process. These simple but cost-effective biosensing strategies all have the potential to be utilized as POC-based biosensors at field level and within applications in resource-limited settings.

### Sensitivity and Selectivity of Nanozymes-Based Biosensors

**Fig. 8:**
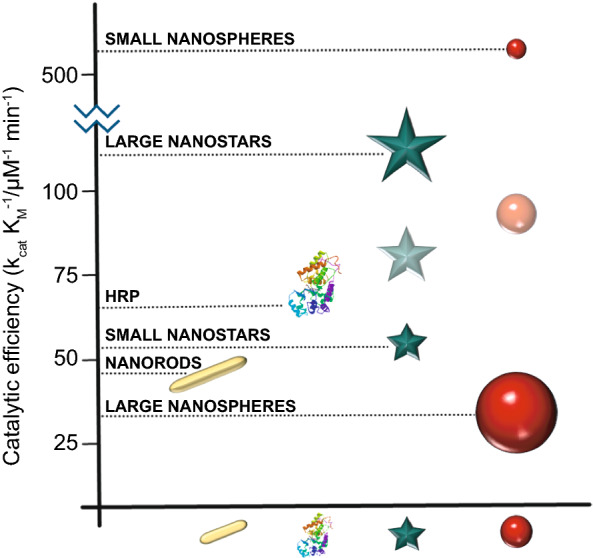
Comparison of the catalytic efficiency for the peroxidase-like activity of different-shaped and different-sized AuNPs and natural enzyme (HRP) [212]. Copyright 2020 Javier Lou-Franco et al.

Apart from the affordability (i.e. low fabrication and detection costs) as an imperative and desirable quality, POC biosensors should possess selectivity (the capacity to respond to a distinctive analyte or parameter without getting affected by the physiological interferences) and sensitivity (the ability to distinguish among comparable values) towards the analyte [184]. Although many studies have been reported for the development of biosensing devices for potential POC applications [206–208], however the main drawbacks of the existing technologies usually are either (i) lower sensitivity, (ii) poor limit of detection, (iii) qualitative or semi-quantitative analysis, or (iv) all these drawbacks combined. To overcome the disadvantages, numerous studies have been carried out for the development of highly sensitive and accurate biosensing platforms with the application of enzyme-mimetic nanoparticles [209]. It is well known that enzyme-mimetic nanoparticles can be available in broad range of shapes and sizes. Along with that, the presence of high surface energy at nanoscale level and suitability for surface modifications with specific ligands made enzyme-mimetic nanoparticles such as metal, metal oxide, MOFs and inorganic nanoparticles a promising candidate for biosensing applications. Moreover, plasmonic nanomaterials with high surface electron density that endow enhanced catalytic activity have been used to improve the sensitivity and limit of detection of target analytes in biosensors for POC-based applications [20]. For example, Au nanozymes with ultrahigh extinction coefficient [210, 211] have been utilized as a key signal amplification component for the development of colorimetric biosensors [20]. It is worth mentioning the catalytic efficiency of Au nanozymes majorly depends on their size, shape, and compositions. A recently published article by our group systematically reviewed the factors affecting the catalytic efficiency of Au nanozymes for their biomedical applications [212]. It has been reported that smaller Au NPs generally have better catalytic properties due to the larger population of low-coordinated gold atoms (corner sites) in nanomaterials resulting in improved catalytic activity [213–215]. These findings also suggested that the presence of electron-rich gold plane at the interface of Au NPs with size of 2 nm or a height of six atomic monolayers attributed to the catalytic activity of Au nanozymes for CO oxidation [215]. These size-dependent catalytic activities could be explained from the perspective of thermodynamic principles where increase in the adsorption free energy with decreasing size led to higher catalytic performance [212]. Nevertheless, this size-dependence rule cannot be generalized to Au NPs with exclusive shapes, notwithstanding being true for nanospheres. There are very few evidences reported to support this statement; for example, Biswas et al. demonstrated the higher catalytic efficiency of Au nanorods of 2.8 aspect ratio marginally higher than that of Au nanospheres of 34 nm [216]. Conversely, McVey et al. found that smaller Au nanospheres (14 nm diameter) showed higher catalytic efficiency in comparison with other models [217]. Moreover, these smaller nanozymes, specifically Au spherical nanozymes, have overcome the efficiency of biological enzymes (Fig. 8) which have the potential to be used in biosensing applications.

**Fig. 9:**
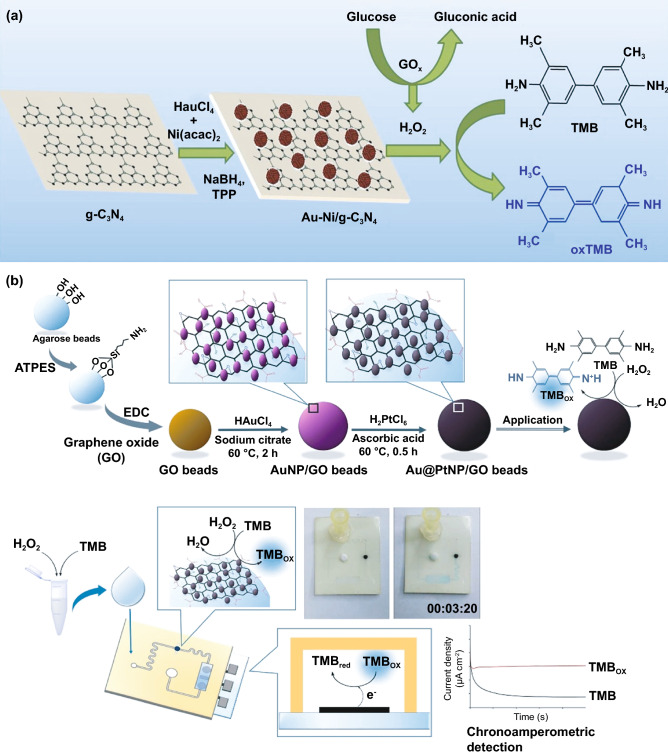
**a** Schematic illustration for the preparation of nanohybrid system consisting Au–Ni (nickel)-based bimetallic nanoparticles doped in graphite carbon nitride sheets and its application for the selective and sensitive detection of glucose [218]. Copyright 2019 Elsevier B.V.** b** Schematic representation of Au@PtNP/GO microbeads preparation and Au@PtNP/GO nanozyme-based electrochemical POC device for quantitative detection of H_2_O_2_ [120]. Copyright 2019 Elsevier

It has been observed that despite having high intrinsic enzyme-mimicking activity of gold nanozymes utilized in biosensing applications, there is a lack of evidence of demonstrating the application of naked Au nanozymes (without any modifications) for the sensitive and specific biosensor development, which could be potentially used in POC-based applications following the ASSURED criteria. Thus, a hybrid system based on Au nanozymes with synergistically enhanced catalytic properties could be a promising approach to address the issue. A good example of this approach is Au–Ni-based bimetallic nanoparticles doped in graphite carbon nitride sheets nanohybrid system (Au–Ni/g-C_3_N_4_) fabricated by Darabdhara and co-workers for the sensitive detection of glucose in serum samples [218] (Fig. 9a). The bimetallic nanocomposite (Au–Ni/g-C_3_N_4_) demonstrated enhanced peroxidase-like activity compared to their monometallic (Au/g-C_3_N_4_; Ni/g-C_3_N_4_) counter parts. The kinetic study revealed the lower *K*_*m*_ value of nanohybrid composites at optimized conditions (pH: 4, 35 ℃), attributing higher affinity towards peroxidase substrate (TMB) compared to the monometallic nanoparticles and natural enzymes (HRP). The lower *K*_*m*_ value attributing higher affinity indicates that for intrinsic peroxidase-like activity of nanozyme within a lower substrate concentration the reaction velocity could reach up to half of the maximum velocity. The synergistic effect accounts for the suitable electronic charge transfer between Ni and Au, resulting in higher electron charge density on Au followed by enhanced TMB oxidation rate. Furthermore, using this enhanced catalytic property of nanohybrid system incorporated with GOx enzyme, a highly sensitive colorimetric biosensing assay was developed to detect glucose in serum samples with a linear range of 0.5–30 µM and a detection limit of 1.7 µM, which could be applicable for POC-based glucose-biosensing applications [218]. A similar strategy of enhanced catalytic activity of bimetallic nanohybrid system was established by Ko et al. [120]. They demonstrated the enhanced peroxidase-like activity of Au@Pt nanohybrid system with a smaller *K*_*m*_ (0.0075 mM) value, which is 58 times lower than the *K*_*m*_ (0.434 mM) value of HRP towards TMB substrate [50]. Additionally, the nanohybrid system showed lower *K*_*m*_ value (0.138 mM) for H_2_O_2_ when compared with HRP (3.70 mM). As a result, a lower concentration of H_2_O_2_ may be utilized to achieve the maximum catalytic activity of the nanozyme-modified microbeads. These results indicated strong affinity of Au@Pt nanohybrid towards TMB and H_2_O_2_ (substrate), which could be employed in biosensing applications as an alternative to natural enzymes (e.g. HRP) [50]. Based on these findings, they further fabricated an electrochemical POC biosensor using Au@PtNP/GO (graphene oxide) microbeads where GO microbeads coated with Au @Pt nanohybrid exhibited enhanced peroxidase-like activity resulting in highly accurate H_2_O_2_ sensing with a linear range of 1 µM–3 mM and a LOD of 1.62 µM, with 1 min reaction time (Fig. 9b) [120].

**Fig. 10:**
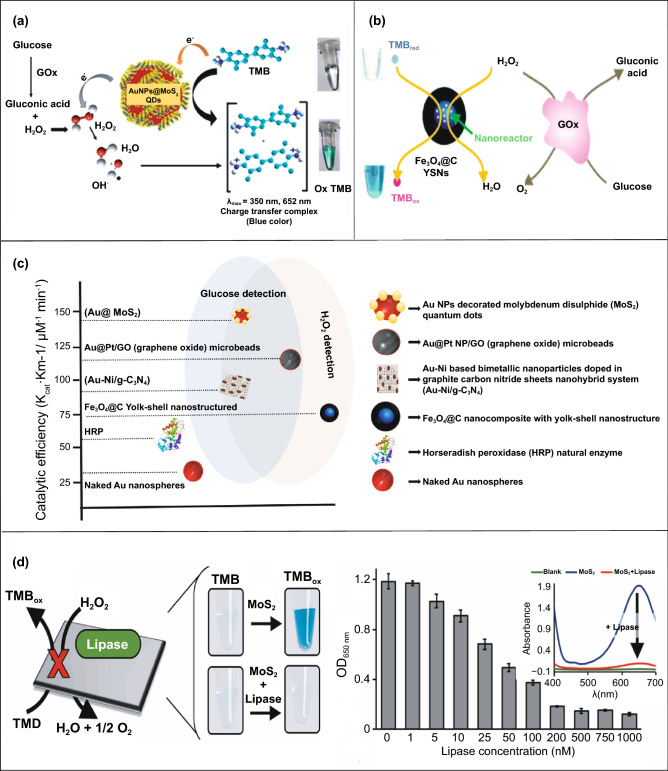
**a** AuNPs@MoS_2_-QDs composite assisted catalytic oxidation of TMB in the presence of H_2_O_2_ and UV–Vis spectra of colorimetric sensing of glucose [219]. Copyright 2018 Elsevier. **b** Analysis of sensitivity and selectivity of glucose biosensing using Fe_3_O_4_@C yolk–shell nanostructured nanozymes. Schematic demonstration of simple and label-free colorimetric biosensing of glucose using Fe_3_O_4_@C nanocomposites. It shows the dose responsive curve, linear calibration curve and selectivity of the glucose detection assay [135]. Copyright 2017 Royal Society of Chemistry. **c** Enhanced catalytic rate/catalytic efficiency (peroxidase-like activity) of nanozymes dependence on different composition and shapes. **d** Schematic illustration and visual demonstration of inhibition of peroxidase-like activity of MoS_2_ nanozyme in the presence of different concentrations of lipase. Inset demonstrate the enzyme-mimicking activity of MoS_2_ with or without lipase [158]. Copyright 2018 Wiley–VCH

Another interesting composition of nanohybrid system with enhanced catalytic activity was reported by Vinita and co-workers [219]. They demonstrated simple one-step synthesis of Au NPs decorated molybdenum disulphide (MoS_2_) quantum dots (Au@ MoS_2_), exhibiting enhanced peroxidase-like activity for the sensitive detection of H_2_O_2_ and glucose [219]. Their results showed a higher affinity of Au@ MoS_2_ towards TMB in comparison with HRP, with an increased catalytic rate (Fig. 10a). The enhanced peroxidase-like activity of the nanohybrid system ensued due to the improved electron transfer efficacy of P-type semiconductor leading to an increased electron density on composites. Electrochemical biosensor based on the enhanced catalytic activity of Au@ MoS_2_ for glucose detection in human fluid samples showed an improved LOD of 68 nM compared to the aforementioned glucose-biosensing assay using Au–Ni/g–C_3_N_4-_GOx nanohybrid [218] system_._ These findings show the significance of compositions of nanomaterials, which play an immense role in modulating the surface electron density resulting in enhanced catalytic activities of nanozymes. Interestingly, the improved catalytic efficiency of nanozymes depending on the shape and structure has also been reported by Wang’s group, where Fe_3_O_4_@C nanocomposite with yolk–shell nanostructure showed enhanced peroxidase-mimicking activity (Fig. 10b) [135]. The hollow space between core and shell acted as a nanoreactor by providing active sites for the catalytic reactions. Based on this phenomenon, they developed a highly sensitive colorimetric biosensor for the detection of H_2_O_2_ with increased sensitivity (LOD 1.12 µM), eight times higher than other reported H_2_O_2_ sensors [52]. Moreover, these interesting findings propose that based on the size, shape, and composition of the nanomaterials, the catalytic activity of the nanozyme could be modulated significantly. Although there is a lack of further research to explore different shapes, sizes, and compositions to obtain experimental information, in general, smaller nanozymes with larger surface-to-volume ratio with higher surface electron density and suitability of electron charge transfer between bimetallic nanozyme components are expected to show improved catalytic activity. With this strategy, nanozymes that are suitable for the development of highly sensitive biosensors by overcoming the efficiency of natural enzymes can be developed (Fig. 10c). It has long been understood that diverse morphological characteristics such as shape, size, crystallographic structure, pH, temperature, and surface coating of nanozymes with different biological/chemical compounds play an immense role in manipulating their catalytic activities [3, 183]. In most cases, surface modification of the nanozymes for better stability (capping agent) or biorecognition agent (for biosensing specificity) has resulted in the suppression of their catalytic activity. However, based on this reduction of catalytic activity towards specific analytes or surface coatings, several studies have been reported for the development of highly sensitive biosensors with the potential of POC-based applications. For example, Nandu and co-workers have demonstrated a highly sensitive colorimetric biosensor based on the masking effect of the peroxidase-like activity of molybdenum disulphide (MoS_2_) 2D-nanomaterials in the presence of lipase (Fig. 10d). The higher affinity of lipase towards the surface of MoS_2_ nanozyme hinders the interaction between TMB (peroxidase substrate) and MoS_2_ resulting in the inhibition of the TMB oxidation process followed by low catalytic activity. The reduction of catalytic activity was reported to be in the linear range of 5–200 nM lipase concentration and with a LOD of 5 nM (Fig. 10d) [158]. In another study, Li et al. [220] demonstrated the development of glutathione (GSH) biosensor based on the suppression of catalase-like activity of Co_3_O_4_ nanocrystals. In the presence of GSH, the catalytic activity of Co_3_O_4_ nanozyme decreased significantly which resulted in the highly sensitive detection of GSH with a LOD of 500 nM [220]. Likewise, inspired from these findings based on the reduction of peroxidase-like activity of IrO_2_/rGO nanocomposites in the presence of low weight biothiols such as GSH, cystine (Cys) and homocysteine (HcY), a highly sensitive biosensor was developed which further lowered the LOD of GSH to 83 nM with high selectivity [221].

**Fig. 11:**
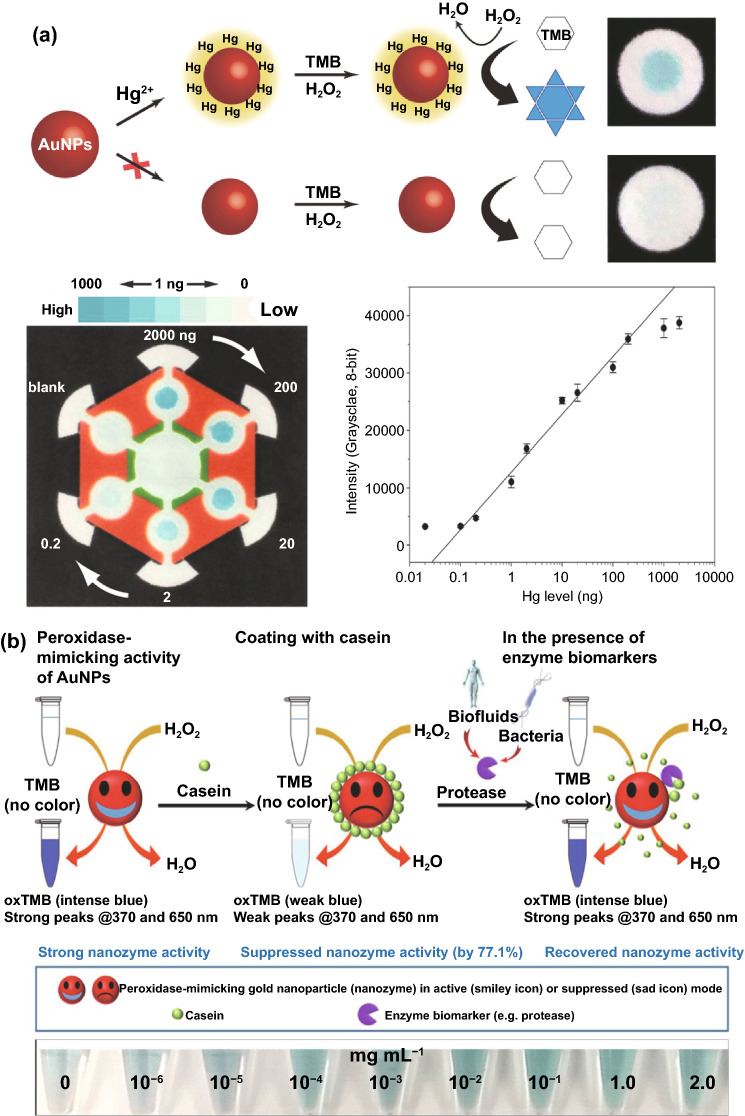
**a** Schematic illustration of the colorimetric biosensing of Hg^2+^ ions using Au nanozyme PAD based on the mercury ion-assisted enhanced catalytic efficiency of Au nanozyme. In the presence of Hg^2+^ ions on the Au nanozyme PAD the catalytic oxidation of TMB enhanced significantly due to the formation of Au–Hg amalgam, resulting in generation of blue colour in the paper-chip. Photograph shows the Au nanozyme PAD with test samples consisting increasing concentration of Hg levels ranging from 0.2–2000 ng. Calibration curve shows the colorimetric response of Au nanozyme PAD-assisted TMB oxidation in the presence of Hg ions [222]. Copyright 2017 Han et al. **b** Colorimetric biosensor for the detection of proteolytic biomarkers based on unusual peroxidase-mimicking activity of Au nanozymes. Picture shows the naked-eye visualization of colorimetric response with the increasing concentration of protease (0–2.0 mg mL^−1^) [217]. Copyright 2018 McVey et al.

Furthermore, the enzyme-mimicking activity could be enhanced in the presence of target analytes. Han and co-workers incorporated Au NPs into a paper-based colorimetric device for the detection of mercury ions (Hg^2+^) in environmental samples (Fig. 11a) [222]. Au NPs showed intrinsic peroxidase-like activity, which could catalyse the oxidation of TMB to generate colorimetric signal. However, in the presence of Hg^2+^ ions, the peroxidase activity enhanced significantly due to the formation of Au–Hg amalgam, which resulted in synergistically enhanced catalytic activity. As a result, an intense colorimetric signal could be generated, and sensitive detection of Hg^2+^ ions could be achieved with a LOD as low as 0.012 ng (1.2 µg L^-1^). By exploiting the suppression and enhancement of the catalytic properties of nanozymes upon exposure to external factors (*e.g.* surface coatings, target analytes, pH, temperature, *etc.*), Cao and co-workers demonstrated that peroxidase-mimicking activity of Au NPs could be exploited for the development of highly sensitive colorimetric approach for the detection of proteolytic biomarkers in real samples (Fig. 11b) [217]. The authors reported that when the Au NPs were coated with casein, their peroxidase-mimicking activity was superseded by 71% as compared to that of naked Au NPs. However, the catalytic activity was restored after the addition of protease, an enzyme that lysates casein. This provided a basis for the detection of protease enzymes, which are an indication of bacterial infection or contamination. In addition, the same group improved the colorimetric detection of Hg^2+^ ions in seawater matrices, by first discovering that the catalytic activity of bare-Au NPs could be diminished by ~74% by their surface coating [223]. When the Au NPs were stabilized with oligo-ethylene glycol (OEG), OEG-Au NPs in their colloidal form presented high stability in high electrolyte concentrations (up to 20%) and across a wide range of pH (1–14). Interestingly, the catalytic activity of OEG–Au NPs for the oxidation of TMB was strongly suppressed by the coating but enhanced upon formation of Au–Hg amalgam. The colorimetric approach provided a LOD of 13 µg L^-1^ in a costal seawater certified reference material (CRM), with a response that could be obtained in less than 45 min [223].

**Fig. 12:**
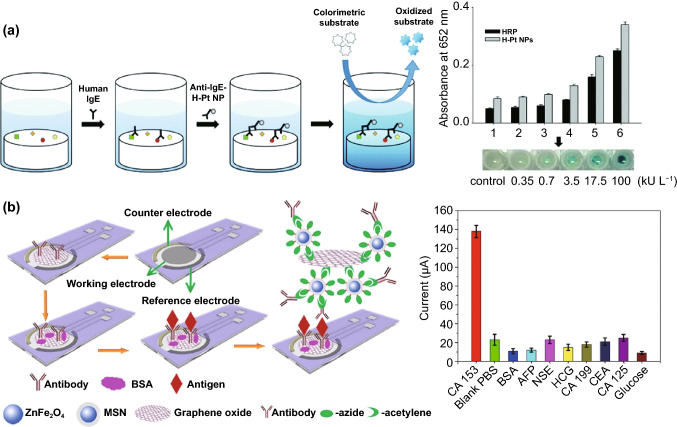
**a** Peroxidase-mimicking activity of H-Pt nanozyme-based ImmunoCAP (laboratory tests for serum allergen-specific IgE antibodies) diagnostic system for the sensitive detection of human IgE [132]. Copyright 2018 Royal Society of Chemistry. **b** Schematic representation of the fabrication of electrochemical immunosensor using magnetic silica NPs/GO nanocomposites for the detection of cancer antigen 153 (CA 153). Specificity assay of the electrochemical immunosensor for the detection of antigen 153 [227]. Copyright 2014 Elsevier

These interesting findings indicate the suitability of these aforementioned nanozyme-based biological and chemical sensing systems for POC applications following ASSURED criteria in terms of sensitivity. However, affordable POC devices with high sensitivity require excellent specificity and selectivity towards its target analyte or else it will not be fit-for-purpose in the ASSURED criteria defined by WHO. In conventional biosensing technologies utilizing natural enzymes as key sensing component could lead to high specificity towards their specific substrate and target analyte as the key mechanism of specificity relies on the “lock and key” principle between the enzyme and the substrate [224]. However, in terms of designing ASSURED-based biosensors which have to be sensitive, selective, and affordable at the same time, utilization of nanozymes instead of natural enzymes as key sensing components could be a promising and suitable approach due to their cost-effective production, biocompatibility, and excellent catalytic efficiency. It is interesting to note that, as discussed in earlier section (Section 3.1) the flexible nature of nanozymes may provide the accessibility to utilize different substrates for the generation of colorimetric, electrochemical, chemiluminescent, and fluorescent signals for affordable biosensor development. Yet, on the other hand, this advantageous phenomenon could lead to less specific biosensors due to the capability of the nanozymes to catalyse for the conversion of a wide range of substrates. To overcome this limitations, the biocompatible nature of nanozymes along with adequacy for surface alteration with various capturing ligands such as antibody, aptamers, biomarkers, biomolecules (Fig. 5) have played an immense role in improving the specificity and selectivity of detections [37, 131, 132, 225]. The specificity based on the target specific ligands attached to the nanozyme surface could allow the development of highly specific and accurate POC biosensors following ASSURED criteria. Cho and co-workers illustrated that they could develop a nanozyme-based colorimetric detection approach based on ImmunoCAP test assay for immunoglobulin E (IgE), a biomarker for allergies [132]. The biosensing assay used hierarchically structured platinum nanoparticles (H-Pt NPs) surface-modified with antibody as a detection and signalling agent (Fig. 12a). The highly selective immunogenic reaction between the target analyte (IgE) and the surface-coated antibody resulted in excellent reaction specificity, while intrinsic peroxidase-like activity of H-Pt nanozymes led to a sensitive detection of total and specific IgE. The sensitivity of the demonstrated biosensor was better than that of conventional ELISA (with HRP-antibody conjugates as detection and signalling probe) (Fig. 12a). Another strategy was reported by Jiang et al. who fabricated immunochromatographic test strip (ITS) utilizing mesoporous Pt-Pd NPs with greatly enhanced peroxidase-like activity for visual and quantitative detection of p53 biomarkers using a hand-held test strip reader [226]. In another study, Ge and co-workers developed disposable electrochemical immunosensor employing peroxidase-like magnetic-silica-graphene oxide (MSN/GO) nanocomposite for the detection of cancer antigen 153. Anti-CA 153 monoclonal antibody immobilized graphene oxide was attached on a screen-printed electrode where magnetic-silica nanoparticles/graphene oxide nanocomposites worked as a signal label due to the elevated peroxidase-like activity. Further specificity assay illustrated the efficacy of anti-CA 153-coated MSN/GO nanozyme-based immunosensors to distinguished target analyte (*i.e.* CA 153) in between possible interferences such as cancer antigen 199 (CA 199), carbohydrate antigen 125, Cinoembryonic antigen (CEA), and bovine serum albumin (BSA) (Fig. 12b). Results demonstrated highly selective and sensitive detection of CA-153 biomarkers [227].

All of these biosensors based on various strategies such as enhancement or masking the catalytic properties of nanozymes with various surface modifications have been employed to develop highly sensitive and accurate colorimetric, electrochemical, or immunosensors which have the potential to be used in POC applications. However, from these observations it is important to note that in few cases of nanozyme-based biosensor development for sensitive and selective detection of a target analyte, there is a requirement for multiple enzyme-assisted catalytic reactions, which lead to utilization of natural enzymes along with nanozymes. For instance, GOx have been used in several glucose biosensors [218, 228, 229] due to their excellent substrate specificity towards glucose which allowed sensitive detection. Henceforth, the question arises in the context of affordability and the potential role of nanozymes in fabricating these biosensors, to be applied in POC testing following ASSURED criteria. Recent progress on the application of multifunctional nanozymes for various biosensors could potentially address our concern in the near future by substituting the role of natural enzymes in the context of affordable POC-based biosensors with excellent specificity and sensitivity.

### Equipment-Free and User-friendly Characteristics of Nanozyme-Based Biosensing Approaches

Another major aspect to be kept in mind while designing POC devices following the ASSURED criteria for in-field biosensing applications is the portability of the devices, without the involvement of sophisticated instrumentation facilities for fabrication or analyte detection [230]. The POC devices with high specificity, sensitivity but based on simple biosensing mechanisms with portability are desired for successful and deliverable sensing applications at resource-limited areas in developing countries. As mentioned in earlier sections, it has been well understood that nanozymes possessing excellent efficiency and flexibility in catalysing various substrates can facilitate production of different signals (colorimetric, electrochemical, chemiluminescent, and fluorescent) which can be measured using various techniques such as naked eye, electrochemical sensor, smartphone app, image analyser, and wearable devices [189, 231].

**Fig. 13:**
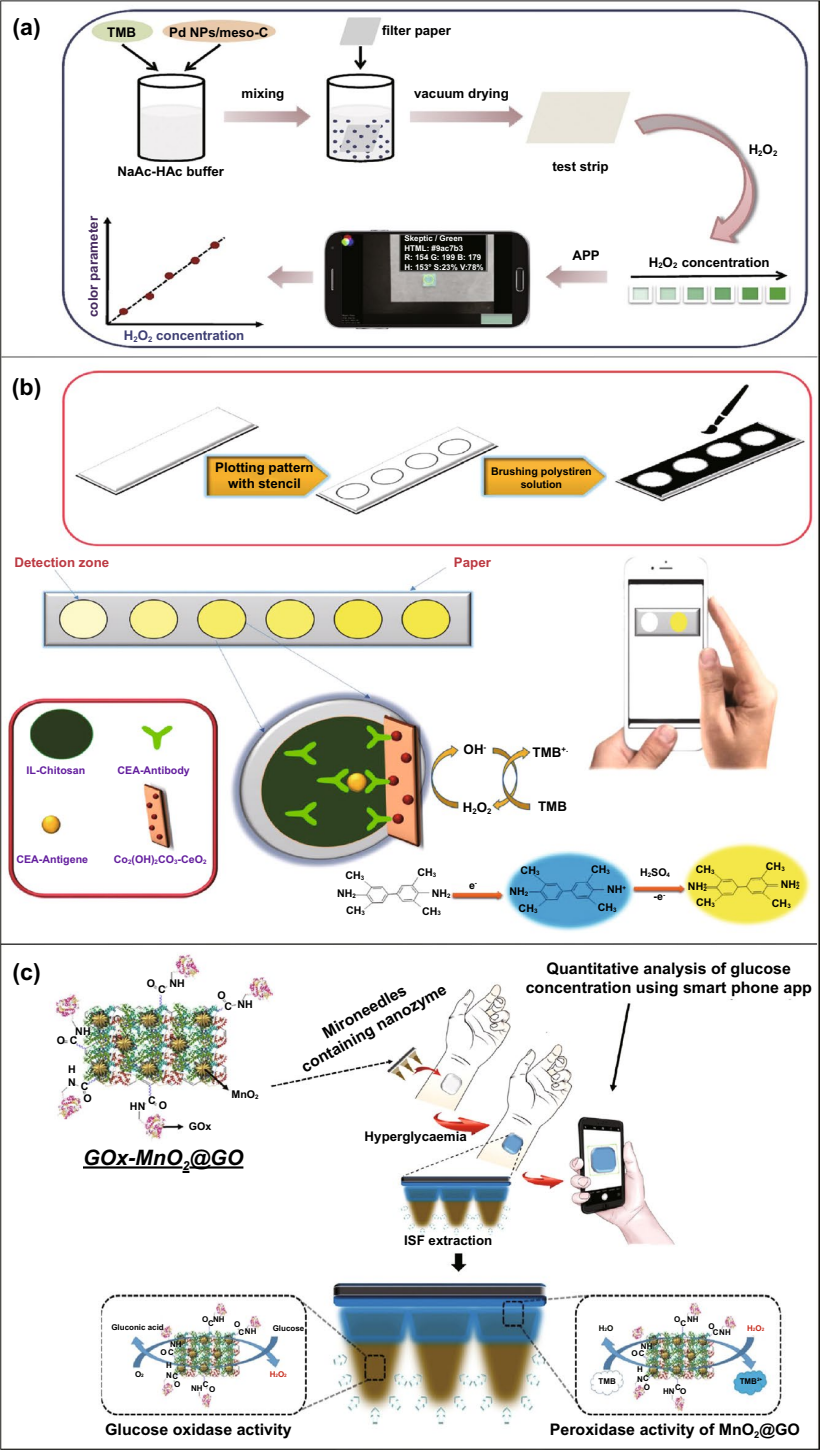
**a** Schematic illustration of preparation of Pd-NPs/meso-C-based test strips and their application in quantitative detection of H_2_O_2_ using smartphone and APP [147]. Copyright 2018 Elsevier.** b** Schematic illustration of the preparation of the µPAD and assay procedure for the sensitive detection of carcinoembryonic antigen (CEA) [153]. Copyright 2018 Elsevier. **c** Schematic illustration of skin interstitial fluid (ISF) extraction using glucose-biosensing microneedle patch (GBMP) and the principle of colorimetric glucose detection using GOx-MnO2@GO as a colorimetric probe and smart phone-based app for quantitative analysis at hyperglycaemic condition [238]. Copyright 2020 Elsevier

The recent developments of portable paper-based analytical devices and straightforward colorimetric biosensing bioassays consisting of enzyme-mimetic nanoparticles have emerged as promising solutions for qualitative and quantitative detection of a wide range of analytes for real-time applications at field level [152, 153]. Among numerous quantitative detection techniques for measurements of signals on paper-based devices, the colorimetric detection technique is more favourable as the nanozyme-assisted enzyme-mimicking catalytic reactions enables the generation of colorimetric signal output with higher intensity, which could be easily visualized by naked eye. Furthermore, applications of a simple and portable device such as smartphone for signal readout in colorimetric POC biosensors have attracted many researchers in recent years [232–235]. Initially, only the camera of simple cell phones was used to capture the assay signal, and further assessment and analysis were still performed on a computer [236]. With the advancement in hardware and software developments, smartphones nowadays become more powerful than ever before, they have much better and stronger processors and operation systems, super resolution camera, and faster Wifi connectivity. These advantages enable them to be an excellent readout device for POC analysis, especially in terms of cost effectiveness, portability, and ease of uses [237]. A suitable example of simple yet sensitive and portable analytical device was demonstrated by Zhang and co-workers, who fabricated a filter paper-based test strip combined with smartphone integrated with user-friendly colour-scanning application (App) for the quantitative detection of H_2_O_2_ in complex sample matrix (Fig. 13a). Mesoporous carbon-dispersed Pd nanoparticles (Pd NPs/meso-C) exhibiting higher peroxidase-like activity triggered the chromogenic reaction of colourless TMB to blue TMB_ox_ in the presence of H_2_O_2_. Pd NPs/meso-C mimic and TMB substrate were immobilized on the filter paper-based test strip, which generated colorimetric signal in the presence of H_2_O_2_. Furthermore, the colour intensity was analysed using a smartphone-based colour-scanning app, which resulted in quantitative H_2_O_2_ detection [147]. Inspired from these interesting findings, Alizadeh and co-workers fabricated a paper-based microfluidic immunosensor for the detection of Carcinoembryonic antigen (CEA, a tumour marker) using Co_2_(OH)_2_CO_3_-CeO_2_ nanocomposites, by a hand-held process without the implementation of sophisticated instrumentation. Secondary antibody (specific to CEA) coated Co_2_(OH)_2_CO_3_-CeO_2_ generates strong colorimetric signals by catalysing TMB in the presence of CEA on the paper strip (Fig. 13b). Furthermore, for quantitative analysis of CEA, a smartphone integrated with colour picker software app was employed to analyse the images, which resulted in sensitive detection of CEA based on the colour intensity generated on the paper-based microfluidics platform [153]. Based on the similar strategy, the same group have developed a paper-based portable biosensor integrated with smartphone for quantitative and qualitative analysis of glucose [152]. They demonstrated the application of Co_3_O_4_-CeO_2_ nanozyme composites possessing higher peroxidase-like activity than that of natural enzyme (HRP), which could catalyse the oxidation between H_2_O_2_ (produced from glucose oxidation using glucose oxidase enzyme) and colorimetric substrate TMB, resulting in colour change which can be visualized by the naked eye. Quantitative analysis of glucose in human serum sample by measuring the colour intensity of the catalytic reaction was performed by using a software installed in smartphone. Based on these interesting findings, recent development of a non-invasive instrument-free glucose biosensor was reported by Yang’s group [238]. They fabricated glucose-biosensing microneedle patch (GBMP) consisting of GOx-conjugated MnO_2_/ graphene oxide nanozymes (GOx-MnO_2_@GO) and swelling methacrylate gelatin (MeGel). In hyperglycaemic condition, once being inserted into the skin, the interstitial fluid (ISF) was gradually diffused into the GBMP due to the hydro-affinity of the gel. This led to the production of gluconic acid and H_2_O_2_ because of enzymatic reaction of GOx with body glucose. The colorimetric signal was generated due to the peroxidase-like activity of MnO_2@_GO, which catalysed the TMB oxidation in the presence of H_2_O_2_. Finally, using a smartphone app the quantitative analysis was done based on the analysis of the colour intensity of the GBMP (Fig. 13c).

**Fig. 14:**
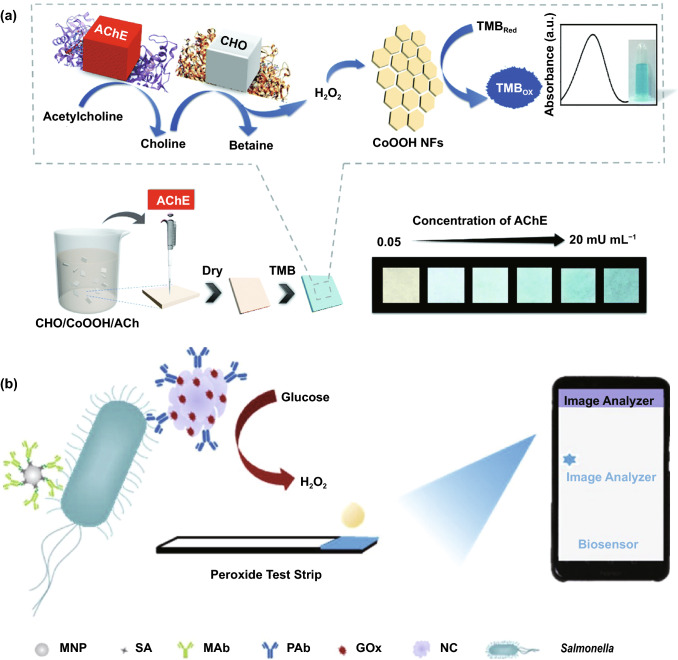
**a** Working mechanism of cobalt oxyhydroxide nanoflakes integrated paper-based point-of-care detection of acetylcholinesterase (AChE). The figure shows fabrication of the paper-based test strips and visual colorimetric detection of AChE based on the colour intensity on the test strips [124]. Copyright 2019 The Royal Society of Chemistry. **b** Schematic representation of the proposed biosensor using magnetic nanoparticles (MNps)-based immunoseparation, nanocluster signal amplification and quantitative detection of *Salmonella typhimurium* using smartphone-based application [239]. Copyright 2019 Elsevier

It has been realized that the development of portable POC biosensing devices following the ASSURED criteria requires a simple yet strong signal amplification system, for instance, colorimetric signal, which could be easily analysed using a smartphone, or any other portable readout devices. The role of nanozymes for the generation of explicit colorimetric signal has been widely accepted. However, utilizations of natural enzymes in combination with nanozymes have been broadly explored by several research groups to enhance the catalytic properties of the multifunctional nanozyme complex systems. For example, cobalt oxyhydroxide nanoflakes (CoOOH NFs) possessing high peroxidase-like activity were utilized in combination with choline oxidase (CHO) to generate colorimetric sensing signal for acetylcholinesterase (AchE) detection (Fig. 14a) [124]. Choline produced by the hydrolysis of acetylcholine (ACh) was furthermore oxidized by CHO to produce H_2_O_2,_ which acts as an electron donor to catalyse the peroxidase-like activity of CoOOH NFs to generate blue TMB_ox_. The resulting colour intensity was analysed using smartphone and image J software, which ensued the sensitive detection of AchE with a detection limit of 33 µU mL^-1^ (Fig. 14a) [124]. In another report, Guo et al. [239] developed a portable biosensor for the detection of *Salmonella typhimurium* using magnetic nanoparticles for immune separation and nanoclusters consisting of calcium and glucose oxidase (GNCs) as signal amplifying components. GNCs interacted with the immune separated target bacteria, which furthermore catalyse the glucose into hydrogen peroxide in a peroxide test strip (Fig. 14b). Smartphone-based APP-assisted analysis of the peroxide test strip resulted in quantitative detection of *Salmonella typhimurium* [239]. Unlike these two above-mentioned biosensing strategies, Cheng et al. utilized Pd–Pt nanozyme composites solely with enhanced peroxidase activity for the development of portable smartphone attached paper strip immunosensor for the simultaneous detection of *Salmonella enteritidis* and *Escherichia coli* O157:H7 [130].

Naked-eye detection is by far the most inexpensive, simplest method and does not require the use of any equipment [240, 241]. However, visual perception may differ between people and lighting conditions may also influence detection; therefore, the use of simple scanners or camera phones for quantification is required. The advantages of using smart devices as signal readout are that they can produce high-resolution images, portability, do not require trained personnel and are widely available all around the world. Even though smartphone applications for biosensing have several advantages, there are a few factors that affect their accuracy and sensitivity. Using smartphone cameras for biosensing applications has certain limitations in terms of high sensitivity or accuracy such as camera pictures are easily distorted and do not correlate well with the real test signals in paper-based systems. Furthermore, smartphone-based assay analysis has largely been based upon grayscale scanning or RGB analysis, and the results are comparable to secondary data; therefore, their accuracy is limited. However, this limitation could be overcome by utilizing Ambient light sensors (ALS)-based technologies to process optical signal.

**Fig. 15:**
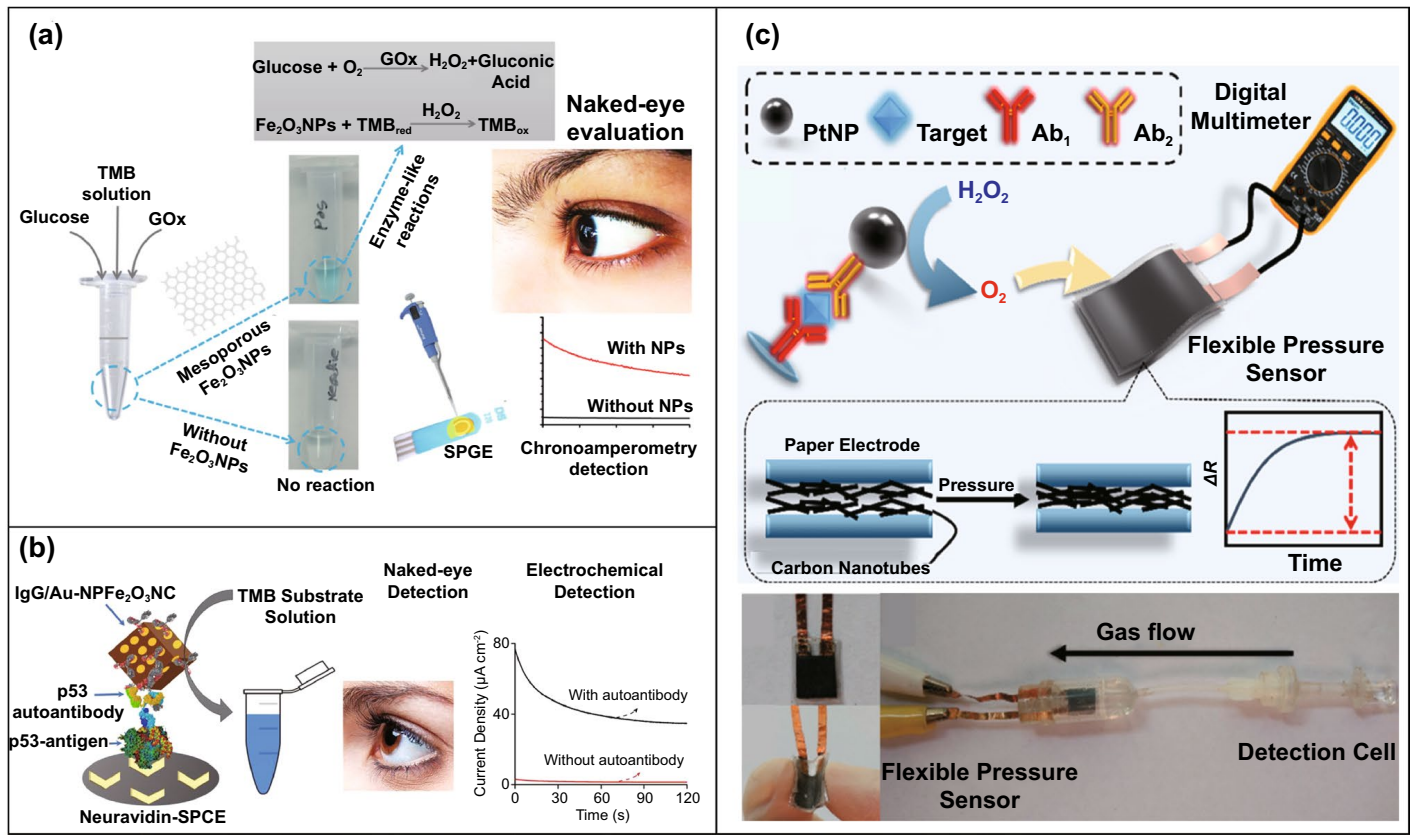
**a** Overview of developed colorimetric and chronoamperometric assay for the detection of glucose using peroxidase-like activity of mesoporous iron oxide nanoparticles [242]. Copyright 2017 American Chemical Society. **b** Schematic representation of the assay for the detection of tumour-associated plasma (and serum) p53 autoantibody. A neutravidin-modified screen-printed carbon electrode was functionalized with biotinylated p53. Serum/plasma samples containing p53-specific autoantibody were then incubated with IgG/Au–NPFe_2_O_3_NC nanocatalysts. Au–NPFe_2_O_3_NC catalysed the oxidation of TMB in the presence of H_2_O_2_ and produced a blue-coloured complex product (naked eye). The level of p53 autoantibody was detected via measuring the intensity (UV–Vis) and amperometric current generated by the product [134]. Copyright 2017 American Chemical Society. **c** Schematic representation of a paper electrode-based flexible pressure sensor for point-of-care immunoassay with digital multimeter readout. Photographs of flexible pressure sensor, bendability of flexible pressure sensor, and immune device based on flexible pressure sensor [165]. Copyright 2018 American Chemical Society

All these discussed biosensors have simple biosensing mechanisms along with user-friendly signal readout set-up, which can be employed as a promising POC devices following ASSURED criteria, where the nanozymes played an imperative role in terms of generation of amplified signal output (mostly colorimetric signals). Typically, these optical techniques are limited by the resolution and focus of the smartphone camera and ambient lighting conditions. Besides, colorimetric signals, electrochemical signals resulted from electrochemical catalytic reactions assisted by nanozymes can also be analysed using simple and portable devices for quantitative detection of target analytes [189]. Meanwhile, electrochemical measurements can still be compared to optical peripherals in terms of their efficacy and are about as independent as possible from the smartphone's capabilities. One excellent example is about glucose biosensing. The rapid evolution of glucose-biosensing technologies in recent years resulted in the successful development of most promising POC devices for glucose detection following all the ASSURED criteria. The basic working principle of electrochemical glucose sensor relies on the oxidation of H_2_O_2_ at the enzyme/nanozyme-modified electrode. One of the major concerns for POC-based electrochemical glucose biosensors development for real sample analysis (i.e. blood, serum) is that the interference of different electro-active biological species such as uric acid, ascorbic acid, 4-acetaminophen (AP), *etc.,* present in the samples*.* However, it could be overcome by applying relatively low potential during the enzymatic reduction of H_2_O_2_ [189]. Recently, based on this strategy, Shiddiky’s group developed a dual mode (electrochemical and colorimetric) glucose biosensor by utilizing peroxidase-mimicking mesoporous Fe_2_O_3_ nanozyme [242]. The electrochemical assay was done using screen-printed gold electrodes and the biosensor was able to detect glucose within a linear range from 1.0 μM to 100 μM with a detection limit of 1.0 μM (Fig. 15a). Portability and user-friendly nature of electrodes used for the development of electrochemical biosensors allowed them for diversified analyte detections. For example, Shiddiky’s group also reported the development of electrochemical immunosensor for p53 autoantibody detection in serum sample by utilizing gold-loaded nanoporous Fe_2_O_3_ nanocubes (Au–NPFe_2_O_3_NC) [134]. The electrochemical quantification was carried out using a new screen-printed carbon electrode. Biotinylated p53 was attached with neutravidin-modified-screen printed electrode based on the strong biotin-neutravidin affinity (Fig. 15b). The electrode was incubated in the serum sample consisting of p53 autoantibody and finally IgG/Au–NPFe_2_O_3_NC was used as detection probe to detect autoantibody present on the electrode.

All of these above-mentioned biosensors were developed based on the electrochemical reactions assisted by nanozymes and the quantitative measurements of the analyte concentration could be achieved by using portable electrodes and digital multimeters. Surprisingly, there have been reports of nanozyme activity being indirectly measured using methods other than electrochemical or colorimetric biosensing techniques, without the use of bulky instrumentation. One excellent example was demonstrated by Yu et al. where catalase-mimicking Pt NPs was employed for the POC-based immune detection of carcinoembryonic antigen (CEA) and the quantitative analysis of the target analyte was performed using a flexible pressure sensor consisting of multiwall carbon nanotubes (Fig. 15c) [165]. Immunoreaction was carried out in a microtiter plate entailing of antibody-modified-Pt nanoparticles (catalase mimic) with a sandwich-type assay. Then due to the catalase-mimicking activity of Pt nanozymes, oxygen (O_2_) produced by the reduction of H_2_O_2_ increased the pressure of the carbon nanotube functionalized electrode, which was then measured with a paper electrode-based flexible pressure light sensor with a digital multimeter readout. The results showed quantitative detection of CEA with a linear range of 0.5-60 ng mL^-1^ and a detection limit of 167 pg mL^-1^, without the involvement of expensive instrumentation [165].

It is worth mentioning that most of the portable POC biosensing systems reported earlier are mostly dependent on the biosensing mechanism based on colorimetric signal amplification. Although there are very few reports available until now which have utilized different biosensing mechanism other than colorimetric signals for portable and equipment-free biosensor development. However, since the fluorescence or optical properties are restricted due to the lack of portability and involvement of sophisticated complex instrumentations, the flexibility of nanozyme applicability can be extended using other sensing mechanisms. We strongly believe that the recent progresses on the miniaturization of large-scale advanced technologies to simple, and portable devices would be able to resolve this concern for future POC-based applications.

### Rapidity and Robustness of Nanozymes-Based Biosensing Approaches

**Fig. 16:**
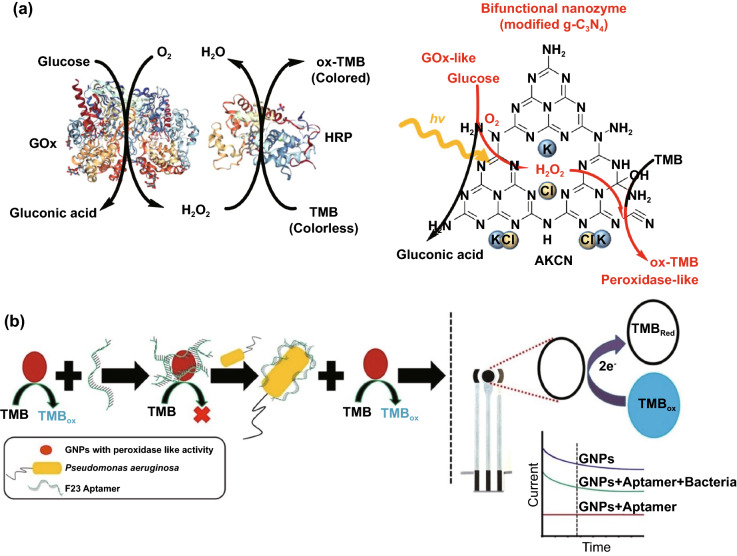
**a** Comparison of two different glucose detection model through cascade reaction. Conventional approach for colorimetric glucose detection using enzyme: glucose oxidase (GO) and horseradish peroxidase (HRP). Glucose detection using a synthetic bifunctional nanozyme (graphitic carbon nitride- g-C3N4: GCN). Photocatalytic aerobic oxidation of glucose leads to the production of H_2_O_2_ further oxidized by bifunctional nanozyme to produce colorimetric signal [246]. Copyright 2019 Nature. **b** Simplified representation of the assay for detection of *Pseudomonas aeruginosa* (PA) using Au nanozymes modified with F-23 aptamer. There is suppression of peroxidase-like activity of Au nanozymes in the presence of F-23 aptamer; however, the activity resumed in the presence of PA. The electrochemical detection of TMB reduction on screen-printed carbon electrode in the presence of PA is shown [247]. Copyright 2019 Springer Nature

Lastly, POC devices designed for on-site applications have to follow two more major key aspects for a successful application, (1) rapid detection of target analytes with high sensitivity and selectivity (2) robust nature of the sensing mechanism with greater stability and maximum reusability [186, 230]. Conventional technologies consisting of natural enzymes for sensing mechanism possesses some limitations in terms of biosensing applicability such as instability, sharp diminution in catalytic activity under harsh conditions and difficulties in reusability, which also make them futile in ASSURED-based POC applications. However, utilization of nanozymes has been considered as an alternative to natural enzymes, resulting in enhanced robustness of the POC devices with the ability of target analytes to be rapidly detected in field-level applications. Biosensing mechanisms with fast responsive signal amplification is the paramount concern for a reliable early detection of target analytes. Through recent progress on the development of biosensors for numerous fields such as, biomedical, environmental, food safety, it has been perceived that rapidity of biosensors majorly relies on the quick cascade of catalytic reactions, resulting in the generation of amplified signal which could be further processed and analysed easily without consuming much time. Nanozymes have been widely considered as an excellent promising candidate to achieve the rapid detection of target analytes due to their upsurged multifunctional enzyme-mimicking catalytic properties considerably more efficient than that of natural enzymes [3, 183]. As discussed in the earlier sections, due to the high surface energy, large surface area and increased electron density of nanozymes this enables the electron transferring suitability between nanozymes and its substrate, resulting in higher affinity for the catalytic reaction with maximum reaction velocity. A brief comparison of catalytic parameters between different nanozymes and natural enzymes utilized in biosensing applications is shown in Table 2. Furthermore, depending on the compositions and other factors such as pH, temperature, surface coatings, the catalytic activity of nanozymes could be modulated to enhance the speed of the reaction kinetics for rapid signal generations. For example, Ding et al. proposed fabrication of *N,N’*-di-carboxy methyl perylene diimides (PDI)-modified Co_3_O_4_ nanocomposites (PDI-Co_3_O_4_) possessing enhanced peroxidase-like activity compared to HRP, for the development of a rapid colorimetric biosensor for H_2_O_2_ and glucose detection [243]. The reaction kinetic analysis revealed that the nanozyme took less time to reach the maximum reaction velocity for TMB oxidation in comparison with HRP, due to the high electron density of the nanozyme after accepting the lone-pair electrons from the amino groups of TMB, which further accelerated the electron transfer between nanozymes and H_2_O_2_, resulting in the rapid catalysis of the oxidation of TMB. Additionally, PDI-Co_3_O_4_ nanocomposites showed *K*_*m*_ value for TMB 10 times lower than that of HRP, suggesting higher sensitivity for the catalytic oxidation with fast response time. Based on this strategy Ding et al. developed a rapid colorimetric detection platform for H_2_O_2_ and glucose within a few minutes, which could be utilized at field-level applications [243]. Furthermore, inspired from these findings the same Liu group (Ding et al.) demonstrated the application of FePt-Au ternary metallic hybrid nanozyme with enhanced catalytic efficiency, for the development of visual colorimetric sensor for the ultrafast detection of H_2_O_2_ with a linear range of 20-700 µM and a LOD of 12.33 µM within 30 s response time [244]. Many other examples of metal-based nanozymes with enhanced catalytic efficiency depending on novel compositions such as Au–Ni/g-C_3_N_4_ [218], Au@Pt [120], Au@ MoS_2_ [219], Fe_3_O_4_@C [135], FeOOH-nitrogen-doped carbon nanocomposites (FN-CNS) [191, 245] have been reported for sensitive and rapid colorimetric detection of H_2_O_2_ and glucose in different sample matrixes. However, most of the sensing strategies utilized natural enzymes (*e.g.* GOx for glucose detection) which also played a major role in enhancing the rate of the catalytic reactions. Therefore, the question of suitability of a rapid biosensing strategy in terms of affordability and robustness of the system arises. Recently, Zhang and co-workers have demonstrated an interesting biosensing strategy based on metal-free nanozyme as a bifunctional oxidase-peroxidase mimic without the involvement of natural enzymes. They have synthesized modified graphite carbon nitride (g-C_3_N_4_: GCN) nanocomposites exhibiting oxidase and peroxidase-like activity both at the same time with enhanced catalytic efficiency than that of HRP (Fig. 16a). The sensing mechanism in microfluidics system with bifunctional cascade catalysis successfully displayed rapid detection of glucose with a detection limit of 0.8 µM within a 30 s response time [246]. Likewise, in case of immunoassay the enhanced catalytic efficiency of nanozymes displayed their ability for rapid detection of target analytes. For example, Au NPs exhibiting high peroxidase-like activity have been utilized for the development of aptamer-mediated colorimetric and electrochemical immunosensor for rapid and sensitive detection of *Pseudomonas aeruginosa* (Fig. 16b). Proposed mechanism (described in Fig. 16b) based on inhibition of peroxidase activity of Au NPs in the presence of F23 (aptamer specific for *Pseudomonas aeruginosa*) resulted in the highly sensitive electrochemical detection of target pathogen with a detection limit of 60 CFU mL^-1^ in water within 10 min [247]. All of these sensing strategies with prompt reaction kinetics could potentially be utilized as rapid POC biosensors for practical applications considerably following the ASSURED criteria established by WHO.

**Table 2:** Comparison of the kinetic parameters of various catalysts utilized in different biosensor developments

Catalyst	Substrate	*K*_*m*_ (mM)	V_max_ (M s^−1^)	Target	Type of biosensor	LOD	Response time	References
VO_2_ nanofibre	TMB	0.518	9.3 × 10^–5^	H_2_O_2_	Colorimetric	0.018 mM	–	[248]
	H_2_O_2_	1.043	4.66 × 10^–4^					
Fe_3_O_4_ MNPS	TMB	0.434	10.00 × 10^–8^	H_2_O_2_	Colorimetric	0.5 µM	–	[249]
	H_2_O_2_	154	9.78 × 10^–8^					
HRP	TMB	0.434	1.24 × 10^–8^	H_2_O_2_	Colorimetric	1 µM	–	[250]
	H_2_O_2_	3.70	2.46 × 10^–8^					
Pt–DNA complexes	H_2_O_2_	–	–	H_2_O_2_	Colorimetric	0.392 mM	10–15 min	[251]
V_2_O_5_ nanozymes	TMB	0.738	1.85 × 10^–5^	H_2_O_2_	Colorimetric	1 µM	–	[252]
	H_2_O_2_	0.232	1.29 × 10^–5^					
PDI–Co_3_O_4_	TMB	0.0562	1.113 × 10^–4^	Glucose	Colorimetric	2.77 µM	3 min	[243]
	H_2_O_2_	34.38	3.924 × 10^–9^					
HRP	TMB	0.434	10.00 × 10^–8^	–	–	–	–	[50]
	H_2_O_2_	3.70	8.71 × 10^–8^					
FePt-Au ternary metallic hybrid nanozyme	TMB	0.445	24.67 × 10^–8^	H_2_O_2_	Colorimetric	12.33 µM	30 s	[244]
	H_2_O_2_	0.0185	0.6894 × 10^–8^					
Graphite carbon nitride (g-C_3_N_4_: GCN) nanocomposites	TMB	0.601	4.22 × 10^–8^	Glucose	Colorimetric	0.8 µM	30 s	[246]
	H_2_O_2_	0.79	6.78 × 10^–8^					
Casein-Au NPs	H_2_O_2_	0.17	1.6 × 10^–8^	Protease	Colorimetric	44 ng mL^−1^	90 min	[217]
HRP	H_2_O_2_	0.079	3 × 10^–10^	–	Colorimetric	–	–	
Pd-Ir NPs@GVs (gold vesicle)	TMB	0.20	1.7 × 10^–7^	PSA	Colorimetric (enzyme-free ELISA)	31 fg mL^−1^	~ 1 h	[253]
HRP	TMB	0.43	1.0 × 10^–7^	PSA	Colorimetric (HRP-ELISA)	48 pg mL^−1^	> ~ 1 h	
Mesoporous iron oxide (Fe_2_O_3_) NPs	TMB	0.153	2.94 × 10^–8^	Glucose	Colorimetric and electrochemical	0.001 mM	–	[242]
	H_2_O_2_	86.425	3.05 × 10^–8^					
Fe_3_O_4_@SiO_2_@Au MNPs	TMB	5.71	1.43 × 10^–7^	Glucose	Colorimetric	3 µM	~ 1 h	[254]
	H_2_O_2_	2.05	60.88 × 10^–7^					
Au@PtNP/GO microbeads	TMB	0.0075	2.659 × 10^–8^	H_2_O_2_	Electrochemical	1.62 µM	1 min	[120]
	H_2_O_2_	0.138	4.815 × 10^–8^

Apart from the rapid nature of the biosensors for POC applications, robustness of the biosensing system remains another important concern where several factors such as pH, temperature, environmental interference, and suitability of the substrate specific reaction mechanism played an immense role for practical applications (Table 3). Amongst several advantages of nanozymes for ASSURED criteria-based POC biosensing applications, the robust nature of the nanozymes has been widely accepted and explored in recent years. Unlike natural enzymes, nanozymes can retain their intrinsic catalytic activity in harsh physiological conditions such as a wide range of pH and temperature, existing numerous examples of this in the literature. One such example was reported by Yan’s group who first discovered the catalytic efficiency of the Fe_3_O_4_ nanozymes at broad range of pH (0–12) and temperature (4–90 ℃), of which the property was further employed for different biosensor developments [50]. Few examples of different biosensors utilizing nanozymes possessing enzyme-mimicking activities within a broad range of pH and temperature variations are summarized in Table 3. Shortly after Yan’s work [50], Wei and Wang developed novel biosensing platforms for the detection of H_2_O_2_ and glucose, both using robust and highly stable nature of Fe_3_O_4_ nanozymes [52]. More importantly, the robust nature of nanozymes allowed them to catalyse different substrates depending on different pH and temperature conditions. For instance, Fe_3_O_4_ nanozymes exhibiting peroxidase-like activity have been reported to catalyse different substrates (e.g. TMB, ABTS, OPD, *etc.*) for the development of H_2_O_2_ biosensors. Based on the multi substrate-catalysing capability, Chang et al. reported the application of this biosensing system for sensitive and selective detection of several ions, ascorbic acid, glucose, *etc.* [255]. Moreover, nanozyme-assisted catalysis of various substrates could result in the generation of catalytic products with either colorimetric, electrochemical or chemiluminescent property. In another study, robustness of enzyme-mimicking Au NPs supported on molybdenum disulphide nanoribon matrix have been utilized for the quantitative analysis of cholesterol in human serum sample. The proposed sensing mechanism depending on the excellent peroxidase-like activity of MoS_2_ NRs-Au NPs resulted in high stability after weeks and retained the ability to perform appreciably in a wide range of pH (3–6) and temperatures (25–60 °C) [256].

**Table 3:** Examples of nanozyme-based biosensors utilizing robust nature of nanozymes capable of possessing catalytic activity at broad range of pH and temperature

Nanozyme/catalyst	Mimicking activity	Target	Type of biosensor	Working condition of nanozyme		Optimal condition for biosensing		References
				pH	Temperature (°C)	pH	Temperature (°C)	
Fe_3_O_4_	Peroxidase	Blood glucose	Colorimetric	2–11	30–80	7.4	30	[260]
GO-Fe_3_O_4_	Peroxidase	Glucose	Colorimetric	3–5	30–50	4	40	[261]
Fe_3_O_4_	Peroxidase	Urinary protein	Fluorescence	–	–	7	37	[262]
γ-Fe_2_O_3_	Peroxidase	H_2_O_2,_ Glucose	Colorimetric	2–8	5–50	5	30	[263]
Au@Pt NR-antigen	Peroxidase	Measles virus	Colorimetric (ELISA)	3–9	20–80	3–9	20–80	[264]
Pt–DNA complexes	Peroxidase	H_2_O_2_	Colorimetric	–	–	5	25	[251]
VS_2_ nanosheets	Peroxidase	Glucose	Colorimetric	2–7	20–80	4	RT	[258]
DNA/MoS_2_ nanosheets	Peroxidase	CEA	Colorimetric	2–5	30–60	4	55	[265]
Single-atom dispersed CNT/FeNC	Peroxidase	Glucose, H_2_O_2_	Colorimetric	2.5–5.5	0–60	3.5	50	[54]
CH-Cu	Laccase-like	Phenolic compounds	Colorimetric	3–9	-18–90	6.8	85	[259]
Natural Laccase enzyme	Laccase	Phenolic compounds	Colorimetric	5–8	-18–60	6.8	40	[259]
CoO-OMC	Peroxidase	Glucose, H_2_O_2_	Colorimetric	3–5	25–50	4.5	37	[266]
Fe_3_O_4_@SiO_2_@Au MNPs	Peroxidase	Glucose,	Colorimetric	2.5–4	50–75	3	70	[254]
HRP	Peroxidase	H_2_O_2_	–	–	–	3.5	40	[50]
Catalase from bovine liver	Catalase	H_2_O_2_	Electrocatalytic	–	–	8	45	[92]
Co_3_O_4_	Catalase	H_2_O_2_	Electrocatalytic	8–10	40–55	> 8–10	> 45	[92]

Another advantage of the robust nature of the nanozyme-based biosensors is reusability. Yang et al. demonstrated an application for Fe_3_O_4_@GOx cross-linked Pt electrode-based electrochemical biosensor for the sensitive detection of glucose, which retained its 83% of responsive efficiency after 52 times uninterrupted detection [257]. Likewise, nanozyme-based immunoassays for the detection of target pathogens have been widely explored for the development of POC devices with highly sensitive, rapid detection with reusability. For example, Cheng and co-workers developed antibody (Ab)-modified Fe-MOF-based colorimetric immunosensor for *Salmonella enteritidis* detection with a detection limit of 34 CFU mL^-1^ in milk samples. The peroxidase-mimicking activity of the Ab-modified Fe-MOF nanoparticles enhanced the signal amplification in the presence of its substrate (TMB). The coefficient of variation (CV) was found to be within 7% after 30 days at 4 °C storage, which was attributed to the robustness and reusability of the immunosensors suitable for POC biosensing at field-level applications [131]. High stability of these nanozymes at extreme conditions such as high pH and temperature endows them to retain their catalytic efficiency, which could be reused after several cycles of catalytic activity for biosensing applications. One such example was reported by Huang et al. where they demonstrated the application of vanadium disulphide (VS_2_) nanosheets as a stable peroxidase mimic for glucose detection [258]. Their study demonstrated the extreme thermally stable nature of VS_2_ nanosheets, which could retain its 90% catalytic efficiency at 80 ℃. Highly stable and robust nature of VS_2_ nanosheets demonstrated their reusability for biosensing applications, which can retain its 85% catalytic efficiency even after 8 times repetitive cycles. In another interesting study, Wang et al. fabricated a new class of nanozyme (denoted as CH–Cu) with laccase mimicking ability, inspired by the active site structure of the laccase enzyme and the electron transfer pathway via the coordination of Cu^+^/Cu^2+^ with a cysteine (C*y*s)–histidine (His) dipeptide [259]. The CH–Cu nanozymes were utilized for the degradation of phenolic compounds and colorimetric detection of epinephrine. Due to the robust nature of the nanozymes, they were able to maintain 80-90% of catalytic activity at harsh pH conditions (pH 3.0 and 9.0) with good thermal stability from -18–90 °C and long-term storage facilities (210 days in air and 20 days in water at 25 °C with 82% and 72 % retained catalytic activity, respectively). The study also suggested that the strong covalent bindings and suitable electron transferring capability through C*y*s-His pathway not only resulted in intrinsic laccase mimicking ability but also assisted the nanozymes to gain its rigidity and regeneration ability through Cu^+^ oxidation pathways in the active site. The recyclability of CH–Cu nanozyme allowed them to retain their 82% relative activity after twelve cycles, while laccase enzymes were unable to be reused. These interesting findings suggest excellent robustness of the nanozymes for their POC-based biosensing applications.

## Conclusion and Outlook

POC-based sensing devices are becoming essential tools for implementation in early diagnosis of the disease biomarkers or infectious pathogens. These advanced and simplified devices are required by not only the clinical diagnosis specialist or the doctor’s office but anyone, even in normal household or outdoors for on-site applications in resource-limited areas. Development of POC devices meeting the ASSURED criteria has been a challenging research area where the device or biosensing system has to be affordable with low fabrication cost, sensitive for detecting analytes at lower concentrations within the border range, and highly specific. Conventional strategies for the fabrication of POC-based biosensing utilizing natural enzymes have several disadvantages, while meeting the ASSURED criteria. Application of nanomaterials possessing enzyme-mimicking activity (nanozymes) integrated in POC-based biosensor devices exhibits several advantages over the use of natural enzymes as major functional components for analyte detection. In this review, several aspects of different nanozymes with various enzyme-mimicking properties for the development of biosensors have been discussed, specifically, how their unique physiochemical and catalytic properties, facile synthesis, robust nature contributed to the development of POC-based biosensing devices, which have the potential to achieve ASSURED criteria. This review summarizes the applications of different types of nanozymes for the development of POC biosensors with the most recent examples of each of the classification of ASSURED criteria. It is evidenced by the aforementioned recent examples, the research field of nanozymes with their suitable biosensing applications has grown sustainably. However, despite this great deal of progress, several research gaps and obstacles still remain in this frontier, which have been identified and need to be tackled to fulfil the great potential of nanozymes in POC-based biosensor development following ASSURED criteria.

First, though many nanomaterials have been subjected for mimicking different natural enzymes however, redox enzyme mimics mostly peroxidase mimics are still dominant for biosensing applications. Very few examples of biosensors have been discussed in this review utilizing enzyme-mimicking phenomenon other than peroxidase (e.g. catalase, SOD, oxidase). Given the variety of natural enzymes, further efforts should be engrossed on developing new strategies on designing nanozymes with new catalytic properties such as synthetase and hydrolase. This could be achieved by combining experimental techniques with computational and/or theoretical strategies for nanozyme designing similar to new functional protein designing. This would not only enhance the flexibility of nanozyme applications in biosensor development but also would endow affordability for a wider range of analyte detection.

Second, the advantages of nanozymes over natural enzymes have been discussed throughout the review where highly stable nature, recyclability, low cost and facile synthesis made nanozymes more approachable for their POC-based biosensor development. However, nanozymes are still lacking behind to compete with natural enzymes in terms of specificity as they could hardly catalyse one specific substrate similar to the natural enzymes. Although the flexible nature of nanozymes for different multisubstrate specificity has provided the platform to develop affordable biosensors at POC settings, still the specificity of the biosensing mechanism would be a paramount concern for successful ASSURED-based biosensor development.

Third, sensitivity of POC-based biosensors mostly rely on the catalytic efficiency of the nanozymes. Few examples have been discussed in this review demonstrating enhanced catalytic activity of nanozymes, which played an immense role in highly sensitive biosensor development. However, to achieve the desired sensitivity and to be classified as ASSURED based POC biosensors, further focus needs to be given towards rational design of high-performance nanozyme. For example, natural enzymes functions together as cluster of enzymes, which provide them the high catalytic efficiency within the confined environment for cascade reactions. Nanozyme assemblies (with different nanozymes, or nanozymes and natural enzymes all together) with higher synergistic effects and improved activities could adopt similar strategy for biosensor development. Here one can argue over the cost effectiveness of the POC-based biosensor development utilizing nanozyme assembly (consisting of nanozyme and natural enzyme all together) in terms of affordability. Further understanding the fundamentals of natural enzyme’s mechanism and designing of engineered multifunctional nanozymes would be a possible way to overcome these challenges. There is only one publication reported so far related to nanozyme having multiple enzyme-mimicking ability (oxidase and peroxidase) with enhanced catalytic activity and could be utilized for the development of POC-based glucose biosensors with potential ASSURED criteria [246].

Fourth, as specificity is one of the most imperative criteria for POC-based biosensor development, surface modification of nanozymes with different target specific ligands (e.g. antibody, aptamers, polymers, biological molecules, etc.) has to be performed to achieve the desired specificity towards target analyte. On the other hand, this could lead to the complete inhibition or suppression of the catalytic properties of the nanozymes. However, due to the tunable nature of nanozyme activities, catalytic properties could be regulated by tailoring the nanozyme compositions (as discussed thoroughly in the review) and by altering the reaction conditions such as pH and temperature. This could overcome the limitations of the applications of nanozymes in a real sample matrix for POC-based biosensing. Finally, inhibition or suppression of the catalytic properties of nanozyme in the presence of specific target analyte or due to their specific surface modification, this phenomenon could also be explored for the development specific and sensitive biosensors with potential POC-based applications (few suitable examples have been discussed in this review). Despite current development, further approaches are needed to deliberately modulate the catalytic properties of nanozymes.

Fifth, it is worth noting that the majority of the previously mentioned portable POC biosensing systems rely on a biosensing mechanism based on colorimetric signal amplification. There have been very few studies to date that have used different biosensing mechanisms rather than colorimetric signals for portable and equipment-free biosensor growth. However, due to the lack of portability and the presence of sophisticated complex instrumentation, the versatility of nanozyme applicability using other sensing methods dependent on fluorescence or optical properties is restricted. We are confident that recent advancements in the miniaturization of large-scale advanced technology to hand-held simple and compact systems would be able to address this issue for potential POC-based applications. It has been clearly demonstrated that nanozymes are strong contenders of the natural counterparts; however, more research efforts need to be paid in order to reach the level of applicability of the natural enzyme system. In addition, because they are nanomaterials mostly produced via bottom-up chemical synthesis routes, it is crucial to improve the reproducibility of nanozymes from batch to batch if they are to be utilized in industrial or clinical settings.

All of the points made in this section, which summarizes the challenges that one may face while designing a nanozyme-based biosensor system for POC applications, are intended to relay important and essential information gleaned from the current state of research rather than to deter future study. Eventually, the success of nanozyme-based POC biosensors lies in the development of user-friendly portable devices with optimized automation, improved shelf-life, and ease-of-use during the data acquisition from on-site field tests in resource-limited areas of developing countries. Scalability, deployment, and disposability has to be kept in mind during the development process for large-scale production of nanozyme-based POC biosensors as a sustainable and achievable solution.

In conclusion, nanozymes have shown promise in advancing various biosensing strategies, which have the potential to be classified as POC-based biosensors under ASSURED guidance due to their several advantages. This study attempted to shed light on the role of nanozymes depending on their unique characteristics for their applicability, potential, and shortcomings of nanozyme-based biosensing strategies. The synergy of nanozymes with numerous biosensing platforms and communication technologies is predicted to yield more innovative and sustainable POC-based biosensors that may benefit research laboratories, to accomplish the ASSURED criteria defined by WHO for diagnostics.
